# Integration of multi-omics data revealed the orphan CpG islands and enhancer-dominated *cis*-regulatory network in glioma

**DOI:** 10.1016/j.isci.2024.110946

**Published:** 2024-09-13

**Authors:** Jiawei Yao, Penglei Yao, Yang Li, Ke He, Xinqi Ma, Qingsong Yang, Junming Jia, Zeren Chen, Shan Yu, Shuqing Gu, Kunliang Chen, Yan Zhao, Weihua Li, Guangzhi Wang, Mian Guo

**Affiliations:** 1 Department of Neurosurgery, The Second Affiliated Hospital of Harbin Medical University, Harbin 150086, China; 2 Department of Pathology, The Second Affiliated Hospital of Harbin Medical University, Harbin 150086, China; 3 Department of Neurosurgery, The First Hospital of Qiqihar, Qiqihar 161005, China; 4 Department of Neurosurgery, People’s Hospital of the Daxing’an Mountain Range, Daxing’an Mountain Range 165300, China; 5 Medical Imaging Department, Shenzhen Second People’s Hospital, the First Affiliated Hospital of Shenzhen University Health Science Center, Shenzhen 518035, China

**Keywords:** Bioinformatics, Medical informatics, Cancer systems biology

## Abstract

The complex transcriptional regulatory network leads to the poor prognosis of glioma. The role of orphan CpG islands (oCGIs) in the transcriptional regulatory network has been overlooked. We conducted a comprehensive exploration of the *cis*-regulatory roles of oCGIs and enhancers by integrating multi-omics data. Direct regulation of target genes by oCGIs or enhancers is of great importance in the *cis*-regulatory network. Furthermore, based on single-cell multi-omics data, we found that the highly activated *cis*-regulatory network in cluster 2 (C2) sustains the high proliferative potential of glioma cells. The upregulation of oCGIs and enhancers related genes in C2 results in glioma patients exhibiting resistance to radiotherapy and chemotherapy. These findings were further validated through glioma cell line related experiments. Our study offers insight into the pathogenesis of glioma and provides a strategy to treat this challenging disease.

## Highlights

• Orphan CpG islands (oCGIs) mediate diverse *cis*-regulatory modes
• oCGIs regulate the tumor microenvironment of glioma
• oCGIs are associated with treatment resistance in glioma patients

## Graphical abstract

**Figure:**
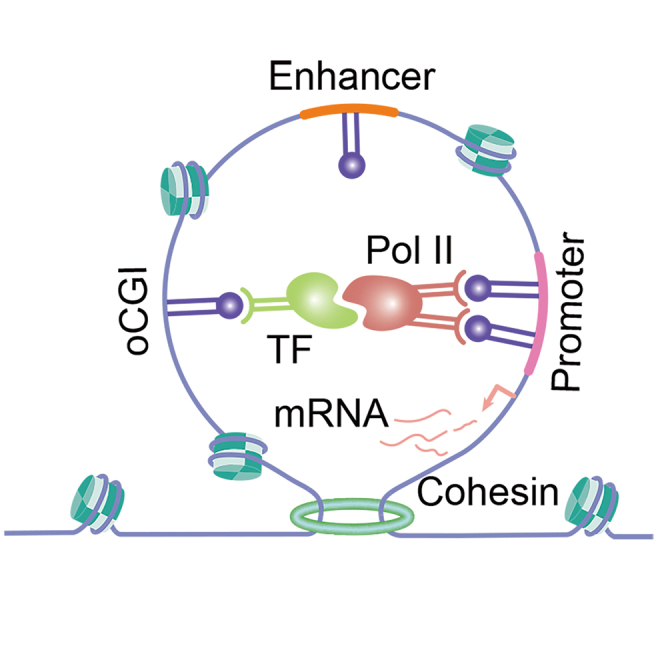

## Introduction

Glioma is the most prevalent primary tumor of the brain and spinal cord, with glioblastoma multiforme (GBM) being the most frequent primary malignant tumor of the brain and central nervous system. It accounts for 14.2% of all tumors and 50.9% of malignant tumors. Despite significant efforts from both basic and clinical researchers, the five-year relative survival rate of glioma patients remains only 35.7%.^1^ The underlying cause of this poor prognosis is the intricate regulatory network of glioma, endowing it with the capacity to adapt to various hostile environments. Elucidating the role of a single target is considerably constrained in this scenario.^2^^,^^3^ With the advancement of sequencing technologies, high-resolution insights have gradually emerged into the development of glioma, paving the way for understanding transcriptional regulation in glioma.^4^^,^^5^

Epigenetic modifications, as a regulatory layer, play a key role in both the upstream and downstream components of the transcriptional network.^6^^,^^7^ Methylation is one of the most prevalent mechanisms regulating transcription.^8^ CpG islands (CGIs) are found in various DNA elements involved in the transcriptional regulatory network, with more than half of promoter regions hosting clustered CGIs.^9^^,^^10^ Furthermore, the presence of CGIs significantly enhances the transcription-activating capacity of enhancers.^11^ Enhancers, as distal *cis*-regulatory elements, are different from proximal regulatory elements such as promoters. They rely on the 3D structure of chromosomes to achieve long-range regulation, thereby playing a pivotal role in the intricate regulatory network of glioma.^12^^,^^13^ Compared to the high density of CGIs in the promoter regions, CGIs are relatively sparse within enhancers. Nevertheless, the function of both is markedly regulated by the methylation levels within their respective CGIs. Increased methylation reduces chromatin accessibility, consequently affecting the binding of enhancers to transcription factors (TFs).^14^^,^^15^ Aberrant epigenetic modifications of CGIs result in transcriptional dysregulation. Abnormal CGIs methylation is closely associated with various diseases, including glioma.^16^^,^^17^ Excluding CGIs located in the classical regulatory regions, the genome still harbors nearly half of orphan CpG islands (oCGIs).^18^ These isolated oCGIs have long been overlooked, and few studies indicated that some oCGIs play an indispensable role in the positive regulatory effect of enhancers located within the same topologically associating domains (TADs).^19^ Not all oCGIs necessarily serve as bridges for enhancer function, and the independent regulatory potential of oCGIs remains uncertain. These aspects remain unknown in the intricate regulatory network of glioma.

In this study, we found that oCGIs act as atypical enhancers, exerting *cis*-regulatory effects and collaboratively regulating target genes in coordination with enhancers, establishing a complex *cis*-transcriptional regulatory network in glioma. Furthermore, single-cell multi-omics data revealed that the *cis*-regulatory role of oCGIs, in conjunction with enhancers, is crucial for maintaining the stemness of glioma cells and is closely associated with various biological processes, such as necrosis and invasion. Additionally, it plays a crucial role in treatment resistance, leading to an adverse prognosis for patients. The *cis*-regulatory role of oCGIs was validated in glioma cell lines. We comprehensively explored potential mechanisms underlying the interaction between oCGIs and enhancers, providing a perspective for unraveling the intricate regulatory network in glioma.

## Results

### Methylation characteristics of oCGIs and enhancers in glioma

We initially identified CGIs in different DNA elements. Consistent with previous findings, the majority of CGIs (78.99%) overlapped with genes or promoter regions, but approximately 20% of oCGIs were dispersed throughout the genome (Figure S1A). These dispersed oCGIs have long been ignored. We performed consensus clustering based on the methylation of oCGIs and enhancers to illuminate the methylation characteristics of oCGIs and classical enhancers. We found that glioma can be broadly classified into two subtypes under various clustering scenarios (Figures S2 and S3). Furthermore, the clustering results based on oCGIs or enhancers divided the glioma samples into two major subtypes, and the two major subtypes obtained from clustering based on oCGIs correspond to the two major subtypes from clustering based on enhancers (Figure S1B). Cluster 1 (C1) was predominantly composed of low-grade glioma (LGG), whereas cluster 2 (C2) primarily included GBM (Figure S1C). Additionally, the isocitrate dehydrogenase (IDH) mutant samples are almost entirely distributed in C1 (Figure S1C; Table S1). We utilized the clustering results based on oCGIs to further explore the role of oCGIs.

### Identifying the *cis*-regulatory network in which oCGIs or enhancers play a dominant role

**Figure 1:**
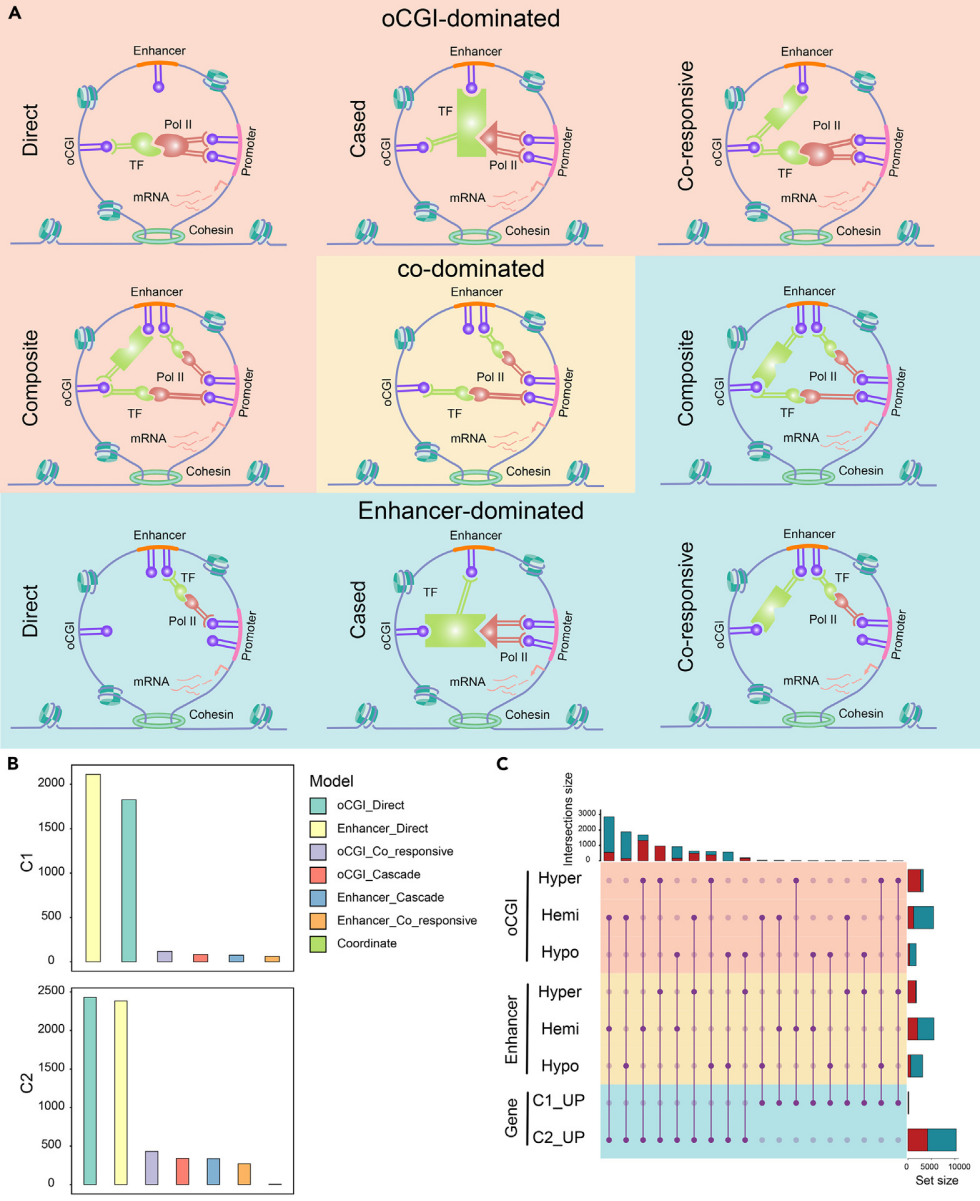
Epigenetic regulatory models in glioma (A) Pattern diagrams for 9 oCGI- and enhancer-dominated *cis*-regulatory models. (B) Composition of epigenetic regulatory models for the two glioma subtypes. (C) Methylation and gene expression levels of components of the epigenetic regulatory model in the two glioma subtypes. In the bar charts in the up and right corner, red represents cluster 1, and blue represents cluster 2. (C1_UP indicates genes upregulated in cluster 1 compared to cluster 2, and C2_UP indicates the opposite).

To elucidate the regulatory patterns among oCGIs, enhancers, and genes, we initially screened combinations with potential regulatory effects based on interactions between oCGI-gene/enhancer-gene pairs in the same TAD. Furthermore, we proposed 9 regulatory models of oCGI-enhancer-gene triplets (Figure 1A). These 9 models elucidated all potential regulatory possibilities, including 4 models where oCGI or enhancer individually played a predominant role (direct, cased, co-responsive, and composite), and 1 model where they were co-dominated (co-dominated). For instance, in the oCGI-dominated direct model, the methylation level of oCGI affected its binding capacity to TFs, thereby affecting the binding of TFs to target gene promoter regions and regulating transcription.^14^^,^^15^ Subsequently, based on Bayesian networks, we determined the most suitable model for each oCGI-enhancer-gene triplet. In addition, models were selected based on the methylation levels of oCGIs and enhancers in conjunction with gene expression. In the end, only 7 models were validated in our dataset (oCGI_Direct, Enhancer_Direct, oCGI_responsive, Enhancer_responsive, oCGI_Cased, Enhancer_Cased, and Coordinate). In both glioma subtypes, the oCGI direct and enhancer direct model predominated, indicating that despite the presence of more complex indirect regulation, oCGIs and enhancers played a dominant role in the direct regulation of target genes. Moreover, among all triplets, the number of triplets with upregulated target genes in C2 is among the highest (Figures 1B and 1C). Furthermore, 1,114 upregulated genes were associated with positive regulation of the tumor (positive regulation of cytokine production and cell migration) (Figure S1D).

**Figure 2:**
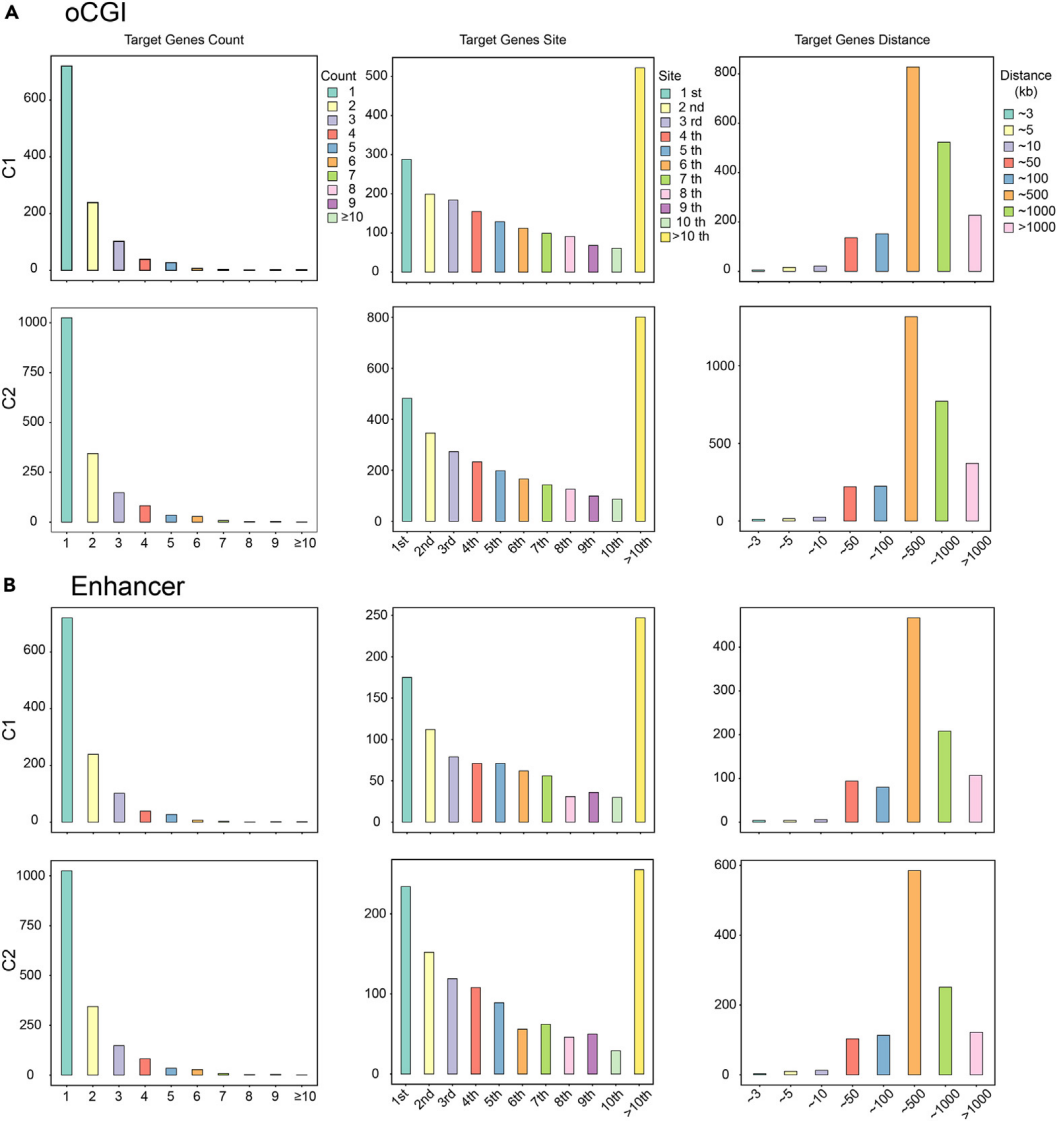
Regulatory characteristics of oCGIs and enhancers (A and B) The number, locations, and distances of target genes were regulated by oCGIs and enhancers in the two glioma subtypes.

oCGIs exhibited similar regulatory characteristics to classical enhancers. Both oCGIs and enhancers can regulate more than one target gene, but the predominant mode of regulation is one-to-one. Secondly, the regulatory effect of oCGIs or enhancers on target genes within the same TAD is not often limited to adjacent loci. On the contrary, non-adjacent regulation models accounted for the majority of the model count. In a significant number of models, oCGIs or enhancers were separated from their target genes by more than 10 genes. Thirdly, the long-range regulatory characteristics of enhancers were also evident in oCGIs. Similar to enhancers, the regulatory distance of oCGIs typically exceeded 500 kb and even surpassed 1,000 kb in some cases. These characteristics were observed in both glioma subtypes (Figures 2A and 2B).

### States of oCGIs and enhancers in the *cis*-regulatory models

Different modes of histone modifications by enhancers serve as markers of their activity. H3K27ac is a marker of active enhancers with positive transcriptional regulatory activity, whereas H3K27me3 is a marker of silenced enhancers. Co-expression of H3K4me1 and H3K27ac indicates enhancers with moderate activity. Promoters also have similar activation and silencing markers. H3K4me3 is significantly enriched in promoter regions as an additional marker.^20^^,^^21^ Due to the lack of chromatin modification data corresponding to The Cancer Genome Atlas (TCGA) samples, we used external samples to determine the mode of chromatin modification of the two glioma subtypes. We first determined to which subtype the external samples belonged. We initially identified the subtypes of 24 glioma samples (GSE121720, GSE121721, GSE189859, and GSE189860) based on distance metrics (Figure S1E). Next, we constructed classifiers for TCGA glioma samples based on oCGI methylation features and gene RNA expression features using 11 machine-learning algorithms. Except for featureless, log_reg, and debug algorithms, other algorithms exhibited excellent discriminatory capabilities for glioma samples in TCGA (Figure S4A). We trained 10 models for each of the eight algorithms based on different resampling results. These models were then applied to the test set samples. Model performance was evaluated using classification error rate and receiver operating characteristic curve (ROC), with cv_glmnet showing excellent performance across all 10 models on both oCGI methylation and RNA expression data. It correctly distinguished the subtypes of all samples when utilizing both types of data (Figures S4A, S4B, and S5).

Next, we analyzed the distribution of different histone modifications across the genome. Both subtypes exhibited similar distribution patterns, with peaks of different histone modifications significantly enriched in gene-related regions (Figure S4C). Furthermore, we characterized the histone modification features in the 3,000 kb regions around all transcription start site (TSS), oCGIs, and enhancers in the two subtypes. C2 exhibited lower methylation levels, higher chromatin accessibility, and higher levels of active promoter and enhancer feature markers, such as H3K27ac peaks, across all regions. All oCGIs displayed histone modification features similar to enhancers (Figure S6A). These findings support their role as “atypical enhancers” with similar functions. Consistent with the dominance of triplets with upregulated target genes in C2, C2 exhibited higher overall transcriptional regulatory activity. Therefore, we focused on genes activated in C2 and repressed in C1 in the follow-up analyses. These genes displayed similar histone modification features between C1 and C2 (Figures S6B and S6C), confirming our previous findings. Finally, combining the regulatory modes obtained from the triplet analysis, the predominant model characteristics in C1 were oCGIs and enhancers with lower activity and higher methylation levels, suppressing gene transcription. In contrast, the dominant regulatory mode in C2 was characterized by active oCGIs and enhancers with lower methylation levels, enabling positive transcriptional regulation (Figures S4D and S4E).

### The *cis*-network in which oCGIs or enhancers play a dominant role in glioma cells

**Figure 3:**
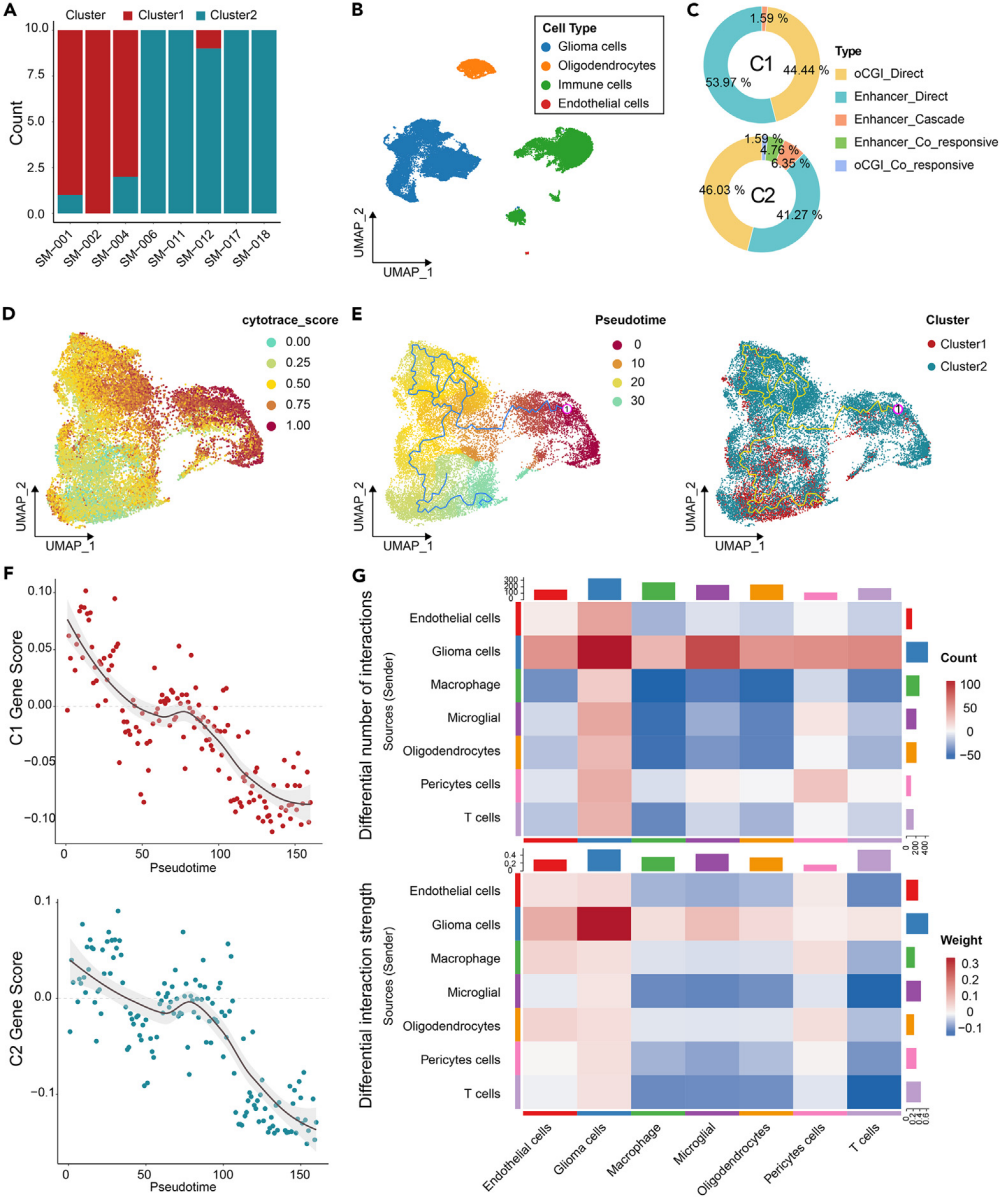
The *cis*-regulatory networks in which oCGIs or enhancers play a dominant role in glioma cells (A) Identification of glioma subtypes using the oCGI RNA classifier with 10 models on 8 glioma samples. (B) Uniform Manifold Approximation and Projection (UMAP) plot depicting the cell types of 8 glioma samples form Verhaak cohort. (C) Composition of the *cis*-regulatory models in which oCGIs or enhancers play a dominant role in glioma cells (top: cluster 1; bottom: cluster 2). (D) Cytotrace scores in glioma cells, where 0 indicates higher differentiation, and 1 indicates lower differentiation. (E) (Left) Differentiation trajectory of tumor cells, with 1 representing the starting point of differentiation. (Right) The connection between cluster 1 and cluster 2 glioma cells with the differentiation trajectory. (F) Changes in the expression of target genes of *cis*-regulatory models in two glioma subtypes along the differentiation trajectory (top: cluster 1; bottom: cluster 2). (G) Cell communication in the tumor microenvironment of the two glioma subtypes. Top: quantity of ligand-receptor pairs between cell types in cluster 1 compared with cluster 2. Bottom: strength of the ligand-receptor pairs between cell types in cluster 1 compared with cluster 2.

Although we have constructed a *cis*-regulatory network based on glioma subtypes, the specific manner in which these regulatory modes function and whether they directly impact tumor cell life cycle remain elusive. Thus, we investigated the regulatory models in which oCGIs or enhancers play a dominant role in tumor cells utilizing single-cell RNA sequencing (scRNA-seq). Our initial approach involved ensuring quality control and cell annotation for scRNA-seq data from 8 samples and filtering cells based on copy number variations, yielding 21,370 tumor cells (Figures 3B and S7A–S7G). Subsequently, we identified subtypes for these 8 samples using a previously developed RNA classifier (Figure 3A) and only retained the tumor cells for subsequent analysis. It should be noted that significant heterogeneity existed among tumor cells from different subtypes (Figures 3E and S7G). Although limited coverage of methylation sites in single-cell reduced representation bisulfite sequencing (scRRBS-seq) prevented identification of regulatory patterns for some triplets, consistent with previous results, oCGI_direct and enhancer_direct modes still primarily regulated both subtypes (Figure 3C). To further characterize the heterogeneity between tumor cells from the two subtypes, we assessed the proliferative potential of tumor cells. Tumor cells in C2 exhibited a significantly higher cytotrace score and a lower degree of differentiation (Figure 3D) (mean cytotrace score: C1: 0.146; C2: 0.637). In line with the results of pseudotime analysis, the tumor cells from C2 primarily aggregated at the initiation of the differentiation trajectory. As cells progressed along the differentiation trajectory, the composition of tumor cells transitioned from C2 cells to C1. Additionally, the upregulated genes in C2 exhibited a significant negative correlation with differentiation time (Figures 3E, 3F, S8A, and S8B; Table S2).

### The mechanism by which the *cis*-regulatory network acts in glioma cells

We initially focused on the communication between various components in the glioma microenvironment to elucidate the precise mechanisms by which the *cis*-regulatory network is involved in glioma cells. Therefore, we needed a more detailed identification of tumor cells. All non-tumor cells were classified into oligodendrocytes, microglia, macrophages, T cells, endothelial cells, and pericytes cells (Figures S8C and S8D). The results of cell communication in the tumor microenvironment showed that interactions between tumor cells and other cells in the tumor microenvironment were stronger in C2 than in C1 (Figure 3G). Many signals favoring tumor cells were significantly activated in C2, such as integrin-related signals (COL9A3 − (ITGA2+ITGB1), JAM3 − (ITGAM+ITGB2), COL6A1 − (ITGA1+ITGB1)), immune suppression signals (CD99 − PILRA), etc. (Figure S8E; Table S3). Furthermore, we explored TFs binding to oCGIs or enhancers. Initially, we scanned the DNA sequences of oCGIs or enhancers, preliminarily selecting TF motifs with binding potential. Subsequently, we identified the upregulated TFs in the two subtypes of tumor cells (Figure S8F; Table S4). By combining the two strategies mentioned previously, we identified TFs potentially regulating different triplets in different subtypes of glioma cells. Based on univariate cox regression and co-expression analysis between oCGIs/enhancers-related TFs and their target genes, we identified key TFs related to the survival of glioma patients (Figure S9A and S9B).

### The effects of the *cis*-network regulatory in different niches of gliomas

**Figure 4:**
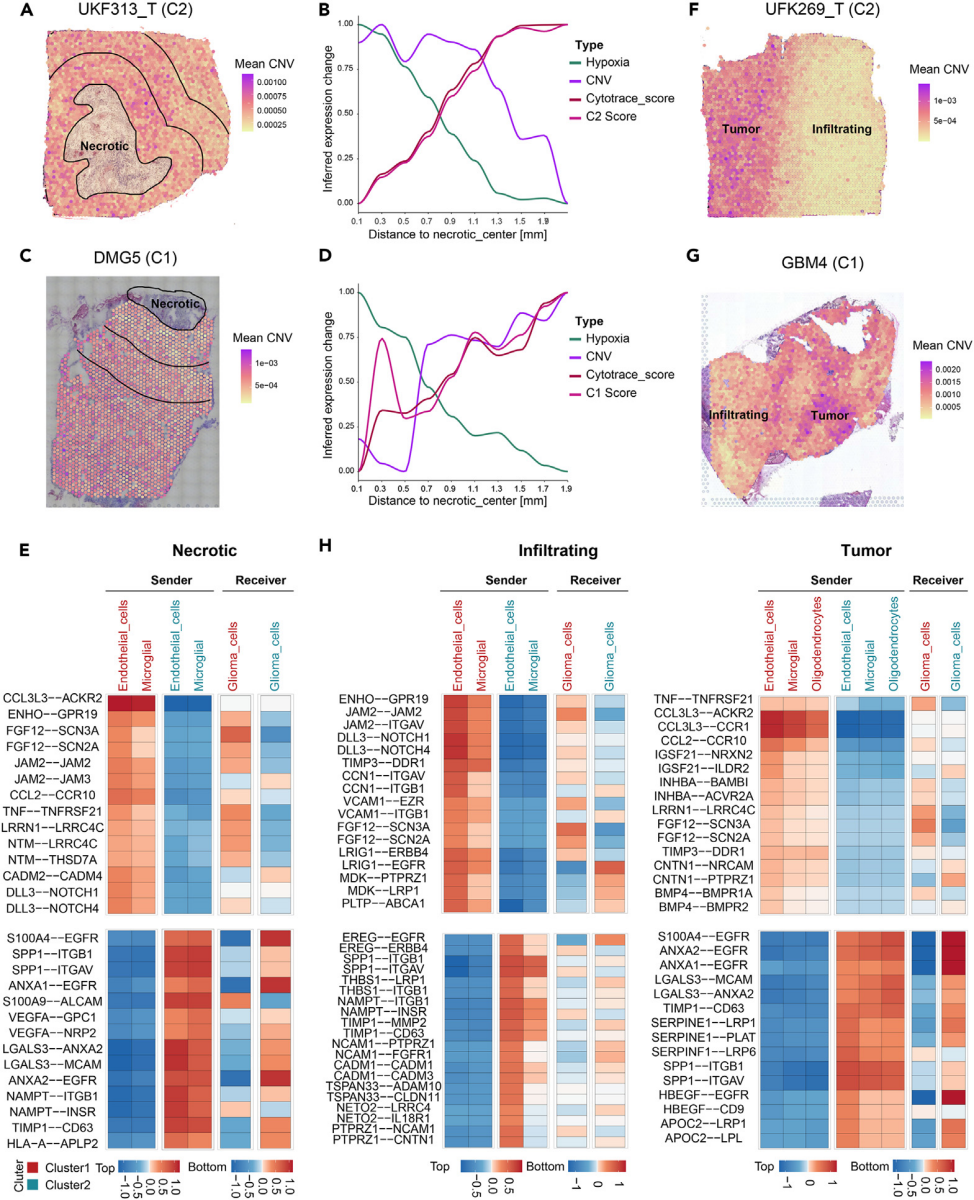
Spatial transcriptomic features of glioma oCGIs-based subtypes (A and C) CNV levels in the necrotic niche of UKF313_T (cluster 2) and DMG5 (cluster 1). (B and D) Expression of hypoxia, CNV, cytotrace score, and *cis* score in the necrotic niche of UKF313_T (cluster 2) and DMG5 (cluster 1) based on the distance from the necrotic center. (E) Cell communication in the necrotic niche of cluster 1 and cluster 2. (F and G) The CNV levels in the infiltrating niche of UKF269_T (cluster 2) and DMG4 (cluster 1). (H) Cell communication in the infiltrating niche of cluster 1 and cluster 2.

Using spatial transcriptomics sequencing (stRNA-seq), we explored the regulatory roles of oCGIs and enhancers in different niches. We first identified the subtypes of glioma (Figure S10A). The necrotic and infiltrating niches of the two subtypes of glioma were analyzed and confirmed by pathologists. The necrotic niche surrounding both subtypes exhibited evident hypoxia and differentiation features. Additionally, genes regulated by oCGIs and enhancers in both clusters were significantly downregulated in this region. Differences in the necrotic niche between the two subtypes were reflected in the copy number variation (CNV) of spots in C2, gradually increasing from the necrotic center toward the outer layer. C1 showed the opposite trend (Figures 4A–4D, S11A, and S11B). To elucidate the reasons leading to this difference, we integrated the 8 scRNA-seq datasets from the aforementioned studies with the respective stRNA-seq dataset for each subtype. Glioma cells mainly co-localized with microglia and endothelial cells, showing stronger colocalization with microglia in regions closer to the necrotic center (Figure S10B and S10C). The necrotic niche in C2 contained a higher proportion of tumor cells, increasing its CNV (Figure S10D). Gene Ontology (GO) analysis showed that MHC-related functions were significantly activated near the necrotic center (layer 1), while membrane protein activity and extracellular matrix activation were more prominent in the outer layer (layer 2), with both subtypes exhibiting similar results (Figure S10E). Cell communication in the necrotic niche revealed a significant overactivity of various chemokines and other immune-related receptor-ligand pairs in C1 (CCL3L3-ACKR2, JAM2-JAM2, CCL2-CCR10, etc.). Similarly, various signals promoting tumor cell activation were more pronounced in C2 (S100A4-EGFR, SPP1-ITGB1, SPP1-ITGAV, etc.) (Figures 4E and S10F; Table S5).

In the infiltrating niche, the CNV, hypoxia, cytotrace score, and *cis* score exhibited similar distributions (*cis* score: the mean expression levels of oCGI/enhancer-related genes upregulated in C1/C2) (Figures 4F, 4G, S11C, and S11D). In the tumor component of the infiltrating niche, tumor cells were predominantly co-localized with oligodendrocytes and endothelial cells, and this distribution pattern was consistent between the two subtypes (Figures S12A and S12B). The tumor region comprised more tumor cells and fewer immune cells compared with the infiltrating region (Figure S12C). The tumor region in C1 activated additional immune-related molecular functions compared with C2 (Figures S12D and S12E). The communication between glioma cells and components of the tumor microenvironment revealed abundant tumor-promoting signals in the infiltrating niches of both subtypes. Immune-related signals were more abundant in C1 compared with C2 (JAM2 − JAM2, CCL3L3 − CCR1, CCL2 − CCR10) (Figures 4H and S12F; Table S6). The gene POLR2L, regulated by oCGI Chr11:728884-729383 in our study, played a significant role in cell communication in C2 glioma cells, although it was not the most significantly activated gene.

### The upregulation of oCGI/enhancer-related genes in C2 is associated with treatment resistance in gliomas

**Figure 5:**
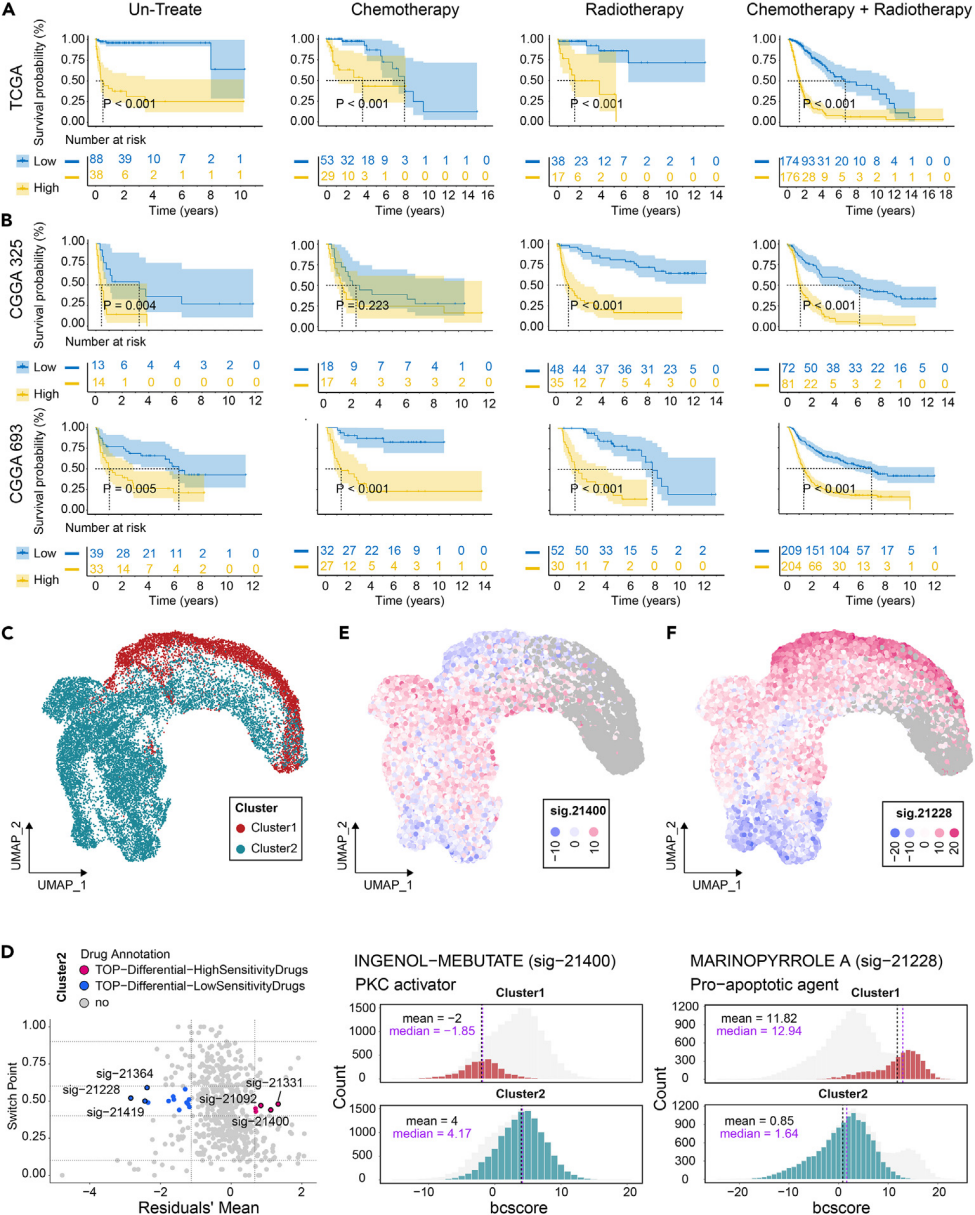
oCGIs/Enhancer-related genes contribute to treatment resistance in glioma patients (A and B) Higher C2 *cis* scores result in shorter overall survival in patients, based on TCGA and CGGA datasets. (C) UMAP plot illustrating the bcscore of glioma cells for two subtypes. (D) Sensitivity of the two subtypes to specific drugs. Data are presented as means or medians of cluster 2 cells. (E and F) UMAP plots of bcscores for ingenol mebutate and marinopyrrole A in the two subtypes (top) and the distribution of bcscores in tumor cells (bottom).

To elucidate the impact of the *cis*-regulatory role of oCGI/enhancers on the clinical treatment of glioma patients, we stratified glioma patients from TCGA into high and low groups based on the mean expression levels of 1,055 oCGI/enhancer-related genes upregulated in C2 (C2 *cis* score). The prognosis of patients with higher C2 *cis* scores was significantly worse under different treatment conditions (untreated, chemotherapy, radiotherapy, and chemotherapy + radiotherapy) (Figure 5A). This pattern of treatment resistance was further validated in the Chinese Glioma Genome Atlas (CGGA) cohorts, including the 325 cohort and 693 cohort (Figure 5B). Clustering results based on drug sensitivity of tumor cells from two subtypes, as shown by beyondcell, revealed significant heterogeneity between the two subtypes (Figure 5C). C2 tumor cells showed higher sensitivity to ingenol mebutate, while C1 tumor cells were more sensitive to marinopyrrole A (Figures 5D–5F).

### Validation of the *cis*-regulatory role of oCGIs

**Figure 6:**
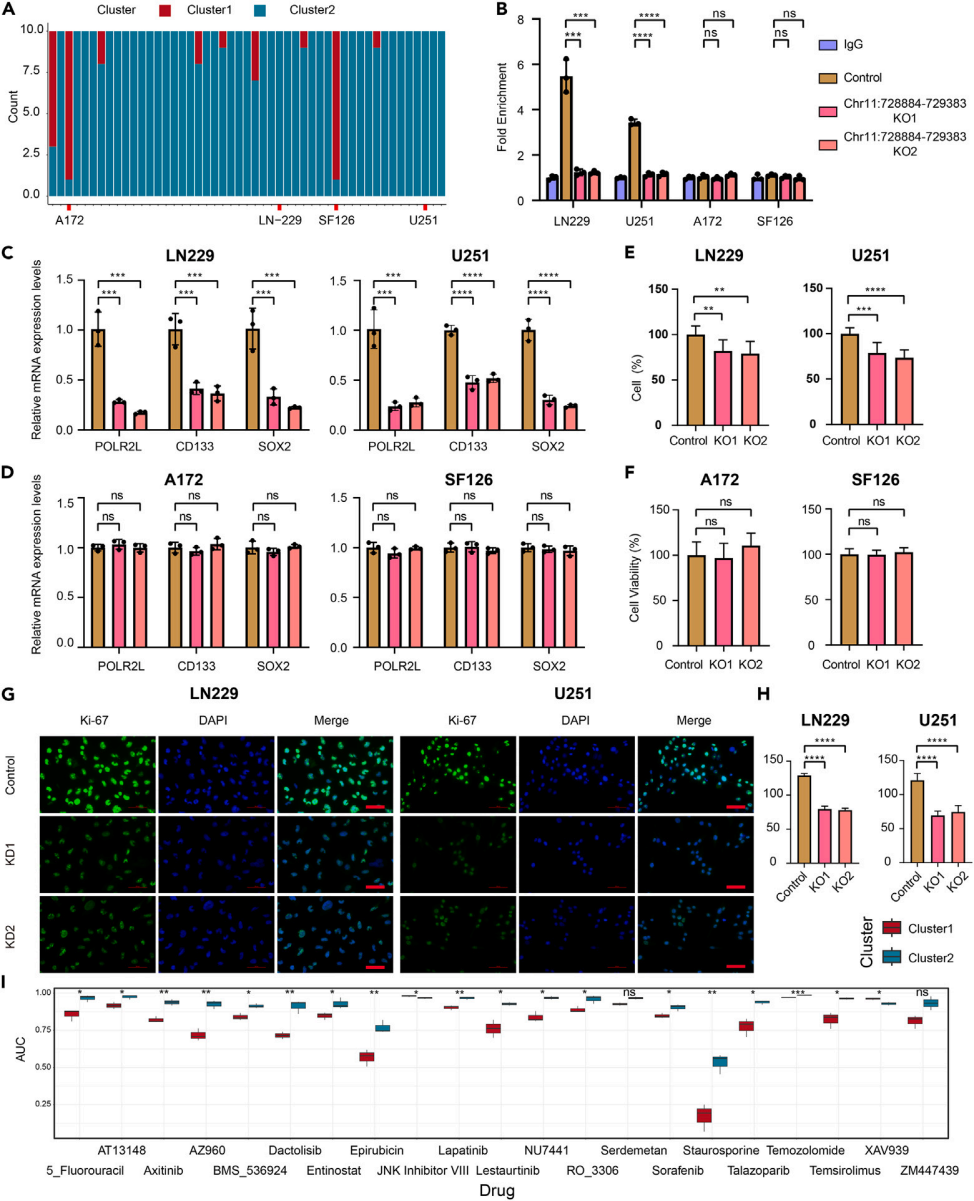
Validation of the *cis*-regulatory effects in which oCGIs play a dominant role (A) Application of the oCGI DNA methylation classifier to 49 glioma cell lines. (B) ChIP qPCR showing significant enrichment of E2F7 in the oCGI Chr11:728884-729383 region in cluster 2 cell lines (LN229 and U251). This enrichment disappeared after oCGI knockout. No such enrichment was observed in cluster 1 cell lines (A172 and SF126). Data are presented as means ± SEM of three independent experiments. (C) In LN229 and U251 cell lines, knockout of oCGI Chr11:728884-729383 decreased the expression of POLR2L, CD133, and SOX2 compared with the control group. Data are presented as means ± SEM of three independent experiments. (D) In A172 and SF126 cell lines, knockout of oCGI Chr11:728884-729383 did not significantly affect the expression of POLR2L, CD133, and SOX2 compared with the control group. Data are presented as means ± SEM of three independent experiments. (E) In LN229 and U251 cell lines, knockout of oCGI Chr11:728884-729383 decreased cell viability compared with the control group. Data are presented as means ± SEM of three independent experiments. (F) In A172 and SF126 cell lines, knockout of oCGI Chr11:728884-729383 did not significantly change cell viability compared with the control group. Data are presented as means ± SEM of three independent experiments. (G and H) In LN229 and U251 cell lines, knockout of oCGI Chr11:728884-729383 resulted in a noticeable attenuation of KI67 (green) compared to the control group. (Blue: nucleus.) Data are presented as means ± SEM of three independent experiments. Scale bar: 100 μm. (I) Drug sensitivity screening of two glioma subtypes cell lines (∗*p* < 0.05, ∗∗*p* < 0.01, ∗∗∗*p* < 0.001, and ∗∗∗∗*p* < 0.0001, *p* was calculated by unpaired t test).

As the *POLR2L* gene plays a crucial role in cell communication between tumor cells and other components of the tumor microenvironment, we selected oCGI (Chr11:728884-729383) and its target, *POLR2L* gene, for validation. Additionally, we identified TF E2F7 as a potential regulatory target based on prognosis analysis and co-expression analysis from the aforementioned studies. First, we identified the subtypes of 49 glioma cell lines based on the DNA methylation data (Figure 6A). Two cell lines from each subtype were chosen for subsequent validation (C1: A172 and SF126; C2: LN229 and U251). The results of chromatin immunoprecipitation (ChIP) combined with qPCR indicated that E2F7 was significantly enriched in the region of oCGI Chr11:728884-729383 in LN229 and U251 cell lines. Additionally, this enrichment disappeared upon the knockout of oCGI Chr11:728884-729383 (the control group is without the knockout of Chr1:728884-729383) (Figure 6B). oCGI Chr11:728884-729383 knockout in LN229 and U251 cell lines markedly decreased the expression of *POLR2L* gene and glioma stem cell-related genes, *CD133* and *SOX2*. However, such a regulatory relationship was not observed in A172 and SF126 cell lines (Figures 6C and 6D). The effect of oCGI Chr11:728884-729383 on cell proliferation was validated in different glioma subtypes through MTT assay and immunofluorescence assay. After oCGI Chr11:728884-729383 knockout, LN229 and U251 cell viability significantly decreased, and the Ki67 fluorescence signal became noticeably weaker compared with the control group (Figures 6E, 6G, and 6H). The effect of oCGI Chr11:728884-729383 knockout on the viability of A172 and SF126 cells was not significant (Figure 6F). To provide better guidance for clinical treatment, we filtered drugs effective for each subtype of glioma. Combining drug sensitivity data from 49 glioma cell lines, we compared the area under the curve (AUC) values for the two glioma subtypes. Apart from JNK inhibitor VIII and XAV939, most anti-tumor drugs were more effective on cell lines from C1 (Figure 6I).

## Discussion

Recent studies have increasingly investigated the regulatory effect of classic enhancers on critical targets in cancer. It is now widely acknowledged that enhancers accelerate oncogenesis.^22^ As such, many researchers paid attention to the pairing of classic enhancers with critical nodes in cancer. However, for highly heterogeneous tumors like glioma, the complexity of the transcriptional regulatory network provides the potential for a broader definition of enhancers. The coordinated transcription-enhancing function of oCGIs establishes a foundation for their non-classical enhancer function.^19^ We constructed the oCGI-enhancer-gene regulatory models to illuminate the importance of oCGIs, which have long been overlooked. We revealed the role of oCGIs as non-classical enhancers in the intricate regulatory network of glioma.

First, the comparative genomic analysis highlighted that oCGIs exhibit DNA methylation characteristics similar to classical enhancers, suggesting that oCGIs can exert transcriptional enhancer functions, either through a mechanism akin to that of classical enhancers or by collaborating with classical enhancers. Furthermore, to elucidate the precise regulatory functions of oCGIs across different glioma samples, we applied the 9 possible models of regulation, encompassing oCGIs-enhancer-gene triplets, based on the DNA methylation features of oCGIs in glioma subtypes. Through mutual information analysis and Bayesian networks, we delineated the regulatory patterns of each triplet. As expected, oCGIs directly or indirectly regulated the target genes. Notably, they exhibited regulatory characteristics similar to classical enhancers, enabling them to regulate several target genes and exert long-range regulation within the same TAD. Although oCGIs and enhancers were primarily engaged in the direct regulation of target genes within the *cis*-regulatory network of glioma, many more complex regulatory models were validated, suggesting that enhancers are potential targets of oCGIs. It enhances our understanding of the *cis*-regulatory network.

The predictive models based on 11 machine-learning algorithms allowed us to accurately identify the glioma subtypes based on their DNA methylation or RNA expression levels. These findings can help integrate data from various sources and comprehensively identify the characteristics of the *cis*-regulatory network. Chromatin modifications serve as markers for identifying the functional states of enhancers or promoters.^20^^,^^21^ The results of chromatin modifications in the two glioma subtypes indicated that tumor cells were more active in C2. In C2, the regulatory models in which oCGIs or enhancers play a dominant role exerted stronger positive transcriptional control through lower methylation levels and higher chromatin accessibility. Therefore, we focused on the major regulators of transcription in C2. Some key factors that were previously overlooked have been revealed in our study, such as *MLX*, whose role in gliomas has never been addressed before (Figures S9A and S9B). In our research, *MLX*, activated as a target gene of C2, emerged as significantly correlated with prognosis and exhibited co-expression with various TFs. The oncogenic role of *MLX* has been reported in osteosarcoma, where enhancer-driven *MLX* expression is upregulated, contributing to metabolic reprogramming.^23^ The *cis*-activation effect in C2 renders patients unresponsive to various clinical treatments, including chemotherapy, radiotherapy, or their combination.

We employed a high-resolution single-cell atlas to further characterize the details of the *cis*-regulatory network in tumor cells. Our findings revealed that tumor cells from C2 typically exhibited higher rates of proliferation. Additionally, the regulatory effects of the regulatory triplets in which oCGIs or enhancers play a dominant role on target genes gradually weakened after differentiation in C2, suggesting that the active *cis*-regulatory network in C2 is a crucial factor for maintaining the heightened activity of tumor cells. Additionally, the communication between tumor cells and the glioma microenvironment was more active in C2 compared with C1, exerting a more pronounced pro-tumor effect.

To elucidate the precise mechanisms by which the *cis*-regulatory network and oCGIs were involved in this complex glioma microenvironment, we characterized different niches by analyzing stRNA-seq. Tumor cells in the center of the tumor exhibited stronger proliferation and invasive capabilities compared with the infiltrating region.^24^ Additionally, due to the vigorous metabolic demands of tumor cells, the tumor center was more susceptible to necrosis. Therefore, we selected the necrotic niche as the tumor center and the infiltrating niche as the tumor periphery. In the necrotic niche of both subtypes, tumor cells were primarily co-localized with microglia and endothelial cells. However, in C2, this co-localization was associated with significantly weaker immune cell infiltration and tumor-activating signals. An increase in co-localization with oligodendrocytes was observed in the infiltrating niche, similar to the signal network in the necrotic niche. C2 showed more pronounced activation of pro-tumor signals. The *cis* activation in C2 is a crucial factor leading to patients’ resistance to various treatment modalities. The *cis* activation in C2 renders patients unable to benefit from various clinical treatments and decreased sensitivity to various drugs.

To explore the regulatory role of oCGIs in the tumor center and periphery, we focused on the common downstream target in cell communication between the two niches, namely the *POLR2L* gene, for subsequent validation. The results indicated that oCGI Chr11:728884-729383 may regulates glioma proliferation by modulating the regulatory effect of TFs E2F7 on the target gene *POLR2L*. Clinical drugs were selected based on distinct oCGIs-based glioma subtypes. Furthermore, our study provided a theoretical foundation for the subsequent development of drugs targeting oCGIs-related targets.

Our study comprehensively explored the role of oCGIs as non-classical enhancers in the transcriptional regulatory network of glioma. Generally, oCGIs directly regulate target genes similar to classical enhancers. In addition, we validated numerous, more intricate models, underscoring the complexity of the regulatory mechanisms of oCGIs in cancer, enabling them to act in a coordinated or regulated manner and significantly affecting glioma progression and treatment resistance. We provided in-depth insights into all potential scenarios within the glioma *cis*-regulatory network, laying a foundation for unraveling the mechanisms behind glioma development.

### Limitations of the study

Although we comprehensively analyzed the *cis*-regulatory network in which oCGIs or enhancers play a dominant role, limitations inherent to single-cell technologies necessitate the validation of only a fraction of the identified models. With the continued advancement of sequencing technologies, we anticipate a more comprehensive understanding of the *cis*-regulatory network based on oCGIs. In addition, our study has the following limitations: First, we used TCGA 450K methylation data, which only covers about 1.5% of CpGs in the human genome. With the popularization of sequencing technologies with higher coverage, such as the Infinium MethylationEPIC BeadChip, it will be beneficial to explore the regulatory roles of oCGIs more comprehensively. Second, we referenced previous studies and used all the enhancers from FANTOM5, which include enhancer information from 432 primary cell samples, 135 tissue samples, and 241 cell line samples from humans. Undoubtedly, more robust conclusions could be drawn based on a comprehensive glioma enhancer atlas. However, currently, most enhancers in gliomas are identified through histone modification peaks rather than CAGE-seq. We believe that CAGE-seq, which identifies eRNA produced by enhancer transcription, is a more accurate method for enhancer localization. Third, we used TADs identified from embryonic brain Hi-C data as the background instead of glioma Hi-C data. Due to different developmental stages, cell types, and even cell states, the 3D chromosomal conformations may vary. Therefore, we conducted a preliminary exploration based on previous studies. In the future, the popularization of high-precision data, such as single-cell Hi-C data, will facilitate further exploration of the regulatory relationships of oCGIs. Finally, the hypermethylation phenomenon caused by the high mutation rate of IDH in LGG may also be a potential key reason for the distribution of oCGIs methylation in different glioma samples. As we discussed in our supplementary results, almost all samples with IDH mutations cluster into one glioma subtype.

## Resource availability

### Lead contact

Further information and requests for resources should be directed to Mian Guo (guomian@hrbmu.edu.cn).

### Materials availability

The study did not generate new unique reagents.

### Data and code availability

• The datasets used during the current study are available from public datasets. The detailed data links are listed in the key resources table.
• This paper does not include original code. We have thoroughly explained the details of all analytical processes in the STAR methods section.
• Any additional information required to reanalyze the data reported in this paper will be shared by the lead upon request.

## Acknowledgments

This work was supported by National Natural Science Foundation of China, China (82173384 and 81773161), 10.13039/501100010877Shenzhen Science and Technology Innovation Commission, China (JCYJ20200109120205924), and Heilongjiang Province Key Research and Development Program, China (2023ZX06C11).

## Author contributions

J.Y.: conceptualization, data curation, formal analysis, methodology, software, visualization, writing – original draft, writing – review & editing; P.Y.: data curation, formal analysis, methodology, software, validation; Y.L.: methodology, resources; K.H.: investigation, software, methodology, validation; X.M.: software, validation; Q.Y.: methodology, visualization; J.J.: writing – original draft; Z.C.: investigation, methodology; S.Y.: Writing – review & editing; S.G.: investigation; K.C.: writing – review & editing; Y.Z.: writing – review & editing; W.L.: funding acquisition, supervision; G.W.: resources, supervision; M.G.: conceptualization, funding acquisition, supervision, project administration, writing – review & editing.

## Declaration of interests

The authors declare no competing interests.

## STAR★Methods

### Key resources table

**Table:** 

REAGENT or RESOURCE	SOURCE	IDENTIFIER
**Antibodies**		

Anti-E2F7 antibody	Abcam	Cat# ab245655; RRID: AB_3224407

**Chemicals, peptides, and recombinant proteins**		

ChIP Assay Kit	Beyotime	Cat# P2078
MTT cell proliferation and cytotoxicity assay kit	Beyotime	Cat# C0009S
Ki67 Cell Proliferation Assay Kit	Beyotime	Cat# C2305S

**Deposited data**		

RNA-seq and DNA methylation data from TCGA	UCSC Xena	https://xena.ucsc.edu/
ATAC-seq data from TCGA	TCGA	https://portal.gdc.cancer.gov/
325 RNA-seq cohort and 693 RNA-seq cohort	CGGA	http://www.cgga.org.cn/
RNA-seq, DNA methylation-seq, and ChIP-seq from 24 samples	GEO	GEO accession: GSE121719, GSE121720, GSE121721, GSE189859, GSE189860, GSE189857
Hi-C data from human embryonic brain	Won et al.^25^	GEO accession: GSE77565
scRNA-seq	Verhaak et al.^26^	https://synapse.org/singlecellglioma
stRNA-seq	GEO	GEO accession: GSE194329
SNP data	the 1000 Genomes Project Phase 3^27^	https://www.internationalgenome.org/phase-3-structural-variant-dataset/
all enhancer information	FANTOM5^28^	https://fantom.gsc.riken.jp/5/datafiles/latest/extra/Enhancers/
DNA methylation data for 49 glioma cell lines	GEO	GEO accession: GSE68379
Drug sensitivity data	GDSC	https://www.cancerrxgene.org/celllines

**Experimental models: Cell lines**		

U251	the Second Affiliated Hospital of Harbin Medical University	N/A
LN229	the Second Affiliated Hospital of Harbin Medical University	N/A
A172	the Second Affiliated Hospital of Harbin Medical University	N/A
SF126	Pricella	Cat# CL-0435

**Software and algorithms**		

R (4.2.3)	R core team	https://www.R-project.org/
R package ConsensusClusterPlus (1.62.0)	Wilkerson et al.^29^	https://bioconductor.org/packages/release/bioc/html/ConsensusClusterPlus.html
R package ggplot2 (3.5.0)	Wickham et al.^30^	https://ggplot2.tidyverse.org/
R package mlr3 (0.17.0)	Lang et al.^31^	https://mlr3.mlr-org.com/
R package Seurat (5.1.0)	Satija et al.^32^	https://satijalab.org/seurat/
R package CytoTRACE (0.3.3)	Gulati et al.^33^	https://cytotrace.stanford.edu/
R package infercnv (0.3.3)	N/A	https://github.com/broadinstitute/infercnv
R package Monocle3 (1.3.1)	N/A	https://github.com/cole-trapnell-lab/monocle3
R package beyondcell (2.2.0)	Maria et al.^34^	https://github.com/cnio-bu/beyondcell
R package SCENIC (1.3.1)	Sara et al.^35^	http://scenic.aertslab.org
R package SPATA2 (2.0.4)	Ravi et al.^4^	https://github.com/theMILOlab/SPATA2
R package CellTrek (0.0.94)	Wei et al.^36^	https://github.com/navinlabcode/CellTrek
R package nichenetr (2.0.4)	Browaeys et al.^37^	https://github.com/saeyslab/nichenetr

### Experimental model and study participant details

#### Cell lines

The glioma cell lines used in this study were obtained from different sources. U251 (originating from a 75-year-old male), LN229 (originating from a 60-year-old female), and A172 (originating from a 53-year-old male) were all provided by the Department of Neurosurgery, the Second Affiliated Hospital of Harbin Medical University. In contrast, the SF126 cell line (originating from a 50-year-old female) was acquired from Pricella (Wuhan, China). Cell lines with oCGI (chr11:728884-729383) knockout were established using the CRISPR/Cas9 system. sgRNAs were designed using Benchling’s CRISPR toolkit (sgRNA1: AGCCCCTTGGAAGAAACGGG; sgRNA2: GGAAGCCCCTTGGAAGAAAC). The knockout process was based on the study by Gong et al.^38^ All cell lines were authenticated by short tandem repeat PCR profiling. Mycoplasma tests were conducted using the Mycoplasma PCR Detection Kit (Beyotime, China), and the results were negative.

### Method details

#### Data collection and quality control

We obtained RNA-seq (703 samples), DNA methylation data (686 samples) and paired clinical information for LGG and GBM from TCGA through the UCSC Xena platform and directly downloaded ATAC-seq data (42 samples) for LGG and GBM from the TCGA. The methylation data we used is from TCGA 450K array, which includes 1.5–1.6% of CpGs in the human genome. The TCGA dataset, used as the primary bulk dataset in this study, provides the basis for all conclusions derived from bulk data unless otherwise specified. The 325 RNA-seq cohort and 693 RNA-seq cohort from the Chinese Glioma Genome Atlas were utilized for the validation of treatment response. Additionally, for validation purposes, we acquired data from 24 samples, including RNA-seq, DNA methylation-seq, and ChIP-seq, from the Gene Expression Omnibus (GEO) database (https://www.ncbi.nlm.nih.gov/geo/, GEO accession: GSE121719, GSE121720, GSE121721, GSE189859, GSE189860, and GSE189857).^39^^,^^40^ For the identification of TADs, we referred to the study by Grabowicz et al., which used Hi-C data from human embryonic brain.^25^^,^^41^ To validate our findings at the single-cell level, we utilized scRNA-seq and scRRBS-seq data from 8 glioma samples provided by Verhaak et al.^26^ Additionally, stRNA-seq data from 29 glioma samples were obtained from the study of Schnell and GSE194329 dataset.^4^^,^^42^ Genomic single nucleotide polymorphism data were obtained from the 1000 Genomes Project Phase 3.^27^ Our study included all enhancer information identified in phase 1 and 2 of FANTOM5.^28^ DNA methylation data for 49 glioma cell lines were obtained from GSE68379. Drug sensitivity data for the cell lines were obtained from the Genomics of Drug Sensitivity in Cancer project (GDSC, https://www.cancerrxgene.org/celllines). All data were analyzed based on the hg19.

#### Identifying glioma subtypes based on DNA methylation levels

We applied the following criteria to quality control TCGA DNA methylation data: (1) Exclusion of probes containing SNPs (99,788 probes were excluded); (2) Exclusion of probes expressed in less than 20% of samples (no probes were excluded); and (3) Exclusion of samples with less than 20% probe expression (44 samples were excluded). We utilized the k-nearest neighbors (KNN) method for imputing missing values in TCGA DNA methylation data. Finally, approximately 0.027% of missing values in the methylation data were imputed. The imputation of missing values using KNN was performed with the impute.knn function from the impute package. We used the default parameters of the function, including K = 10, rowmax = 0.5, and colmax = 0.8. All CGIs are expanded into 500 bp regions (±250 bp), with the CGIs at the center of these regions. If different CGIs have overlapping regions, the overlapping regions are merged into a single region. The region extending 1500 bp upstream and 500 bp downstream of the TSS was defined as the promoter region. CGIs overlapping with promoters and enhancers were removed, and the remaining CGIs were defined as oCGIs.

We conducted consensus clustering of the sample’s oCGIs and enhancer methylation data (average methylation values of the corresponding regions for oCGIs and enhancers). We used the ConsensusClusterPlus package for clustering, defining the number of clusters as 2–20, using the hierarchical algorithm. Select the smallest K value that can effectively distinguish all samples. When clustering enhancer methylation data, K = 7 effectively divided the samples into two main clusters. For oCGIs methylation data, K = 11 effectively divided the samples into two main clusters. Clusters with fewer than 10 samples were discarded to explore the main changes present in gliomas.^29^

#### Construction of the *cis*-regulatory network in which oCGIs or enhancer play a dominant role

We measured the strength of the interaction between oCGIs or enhancers and target genes within the same TADs using mutual information (MI).^43^ The following criteria were employed to construct oCGIs-enhancer-gene triplets targeting the same gene: the MI calculated for oCGIs-gene or enhancer-gene pairs had an adjusted *p*-value of less than 0.05, and any pair with an adjusted *p*-value of greater than 0.05 in either oCGIs-gene or enhancer-gene relationship was filtered out. The preliminary selection of oCGIs-enhancer-gene triplets was analyzed to identify their regulatory patterns using Bayesian networks (https://www.bnlearn.com/). For each triplet, we computed joint probabilities for potential regulatory patterns. For example, the oCGI direct model represented the direct oCGI regulation of target gene expression, while the oCGI cascade model indicated that oCGI regulates the target gene by modulating enhancers. The joint probabilities were calculated as follows:

oCGI-dominated.

oCGI direct: P (O, E, G) = P (O) ∗P (G |O) ∗P (E)
oCGI cascade: P (O, E, G) = P (O) ∗P (E |O) ∗P (G |E)
oCGI co-responsive: P (O, E, G) = P (O) ∗P (E |O) ∗P (G |O)
oCGI composite: P (O, E, G) = P(O) ∗P (E |O) ∗P (G |O: E)

Enhancer-dominated.

Enhancer direct: P (O, E, G) = P (O) ∗P (G |E) ∗P(E)
Enhancer cascade: P (O, E, G) = P (E) ∗P (O |E) ∗P (G |O)
Enhancer co-responsive: P (O, E, G) = P (E) ∗P (O |E) ∗P (G |E)
Enhancer composite: P (O, E, G) = P (E) ∗P (O |E) ∗P (G |O: E)
Co-dominated: P (O, E, G) = P (O) ∗P (E) ∗P (G |O: E)

Where P(O) and P(E) represent the probability distributions of DNA methylation in oCGIs and enhancers, respectively. P (G|O) indicates the conditional probability of gene expression regulated by oCGIs, and P (G|O: E) represents the conditional probability of gene expression regulated simultaneously by both oCGIs and enhancers. The definitions of the other terms are similar to those mentioned above.

We selected the model with the smallest Akaike Information Criterion (AIC) as the regulatory pattern for each triplet. Additionally, we conducted independence testing for each triplet. For example, the *p*-value of independence testing between oCGI-enhancer and enhancer-gene was less than 0.05 for the oCGI direct model of the triplet to be considered valid.

Consistent with conventional understanding, the agreement between DNA methylation level and RNA expression is crucial for model validation. Initially, we defined hypomethylation as β < 0.32 and hypermethylation as β > 0.79 using the β distribution of glioma methylation probe values. Hemimethylation was defined as β values falling within the range of 0.32–0.79 (https://github.com/koyelucd/betaclust). Triplets identified as the CGI direct model in C2, had an oCGI methylation level lower than that in C1 and a gene expression level higher than that in C1. Enrichment analysis was conducted for all upregulated genes in C2.^44^ We employed FIMO to identify potential TF-binding motifs in oCGIs and enhancers (*p* < 0.0001).^45^

#### Construction of glioma classifiers based on multi-omics data of oCGIs

We constructed the classifier using three different methods, which were cross-validated to enhance the credibility of the model. First, we employed the Partitioning Around Medoids (PAM) algorithm to discern the sample allocations based on distance. Next, we incorporated 11 machine learning algorithms, including cv_glmnet, featureless, kknn, lda, log_reg, naive_bayes, ranger, rpart, svm, xgboost, and debug, to construct the models based on RNA and DNA methylation levels. The machine learning algorithms were implemented using the mlr3 package.^31^ RNA-seq and DNA methylation data from 545 TCGA samples were used as the training set, while 24 samples from GEO were employed as the validation dataset. We aligned ChIP-seq data from the validation dataset (H3K27ac, H3K4me3, H3K4me1, and H3K27me3) and ATAC-seq data (42 samples) for LGG and GBM to the corresponding regions of oCGIs, enhancers, and promoters.

#### scRNA-seq and scRRBS-seq data

We applied the previously constructed RNA-seq-based classifier to the bulk data matching the scRNA-seq to identify the sample subtypes. Then, we conducted quality control on 55,284 cells from the 11 glioma samples in the Verhaak cohort. Genes expressed in fewer than 3 cells, cells expressing fewer than 200 genes, cells with a mitochondrial gene percentage exceeding 20% and doublets were filtered out. A total of 47,537 cells were used for the subsequent analysis. The samples were integrated using Harmony.^46^ Tumor cells were annotated using marker genes in conjunction with copy number variations (https://github.com/broadinstitute/infercnv) (Glioma cells: *SOX2, OLG1, GFAP;* Oligodendrocytes: *S100B, MBP;* Immune cells: *PTPRC, ITGAM, CD68, CD4;* Other cells: *VWF, PDGFRB*). Cytotrace was employed to assess the differentiation level of tumor cells.^33^ Monocle3 was used to identify the differentiation trajectories of glioma cells (https://github.com/cole-trapnell-lab/monocle3). SCENIC was utilized to identify significantly upregulated TFs in glioma subtypes.^35^ We applied the same criteria to perform quality control on the scRRBS data. Triplets were identified in tumor cells based on scRNA and scRRBS data. The difference in drug sensitivity between two subtypes of glioma cells was analyzed using the beyondcell package.^34^

#### Spatial transcriptomics data

The stRNA-seq data from 29 glioma samples were initially used to identify glioma subtypes using pseudobulk analysis and then applied the previously trained RNA-seq classifier to the pseudobulk data to identify the stRNA-seq sample subtypes. Subsequently, two pathologists jointly divided the images of stRNA-seq into four regions: vascular, necrotic, cellular, and infiltrating. All stRNA-seq analyses were conducted using SPATA2 package.^4^ Copy number variation was assessed using the runCnvAnalysis function. Images representing different features were visualized using the plotSurfaceComparison function. The CellTrek package was employed to integrate scRNA-seq and stRNA-seq data.^36^ The communication between tumor cells and other components of the tumor microenvironment in different niches was identified using the nichenetr package.^37^ The average expression values of target genes regulated by oCGIs in C1 and C2 are referred to as C1 score and C2 score, respectively.

#### Quantitative PCR with reverse transcription

Total RNA was extracted from glioma cell lines using Trizol (Beyotime, China), and reverse transcription was performed using reverse transcription reagents (RNase H-, RNase inhibitor, and dNTP Mix) (Beyotime, China) following the manufacturer’s instructions. All primers are listed in Table S7 qPCR was performed in 3 times for each of the cell lines U251, LN229, A172, and SF126.

#### Chromatin immunoprecipitation (ChIP)

The ChIP assay was conducted following the protocol outlined in the ChIP assay kit (Beyotime, China). The ChIP assay begins with crosslinking cells with formaldehyde, followed by cell lysis and sonication to shear DNA. The chromatin is then incubated with E2F7 antibody (Abcam, USA) and Protein A + G agarose (ChIP assay kit) for immunoprecipitation. After washing the precipitates with a series of buffers, the crosslinks are reversed and the DNA is purified for analysis. Subsequently, qPCR was employed to assess the immunoprecipitated DNA level. The primers are listed in Table S7. This process was repeated 3 times.

#### MTT assay

In the control group and two oCGI knockout groups (chr11:728884-729383 KO1 and chr11:728884-729383 KO2), 10 μL of MTT (5 mg/mL) (Beyotime, China) was added to each well of a 96-well plate. After 4 h of incubation, 100 μL of formazan (Beyotime, China) was added, and the absorbance was read at 490 nm. This process was repeated 3 times.

#### Immunofluorescence

In brief, coverslips with confluent cells were fixed with 4% paraformaldehyde. Then, immunostaining was performed using KI67 polyclonal antibody (Beyotime, China) and incubated for 2 h. Subsequently, goat anti-rabbit fluorescent secondary antibody (Beyotime, China) was added and incubated for another 2 h. Finally, counterstaining was conducted with DAPI, and the cells were observed under a fluorescence microscope. This process was repeated 3 times.

### Quantification and statistical analysis

Statistics were computed with R software v4.2.2. If not otherwise specified, all referenced packages use default parameters. All molecular experiments were analyzed using unpaired t-tests, with a significance level set at *p* < 0.05. The significance levels were denoted as follows: ∗*p* < 0.05, ∗∗*p* < 0.01, ∗∗∗*p* < 0.001, and ∗∗∗∗*p* < 0.0001, P was calculated by unpaired t-test.

## Supplemental information

Document S1. Figures S1–S12 and Tables S2 and S7

Table S1. The mean expression level of oCGIs, related to Figure 1

Table S3. Cell communication based on scRNA-seq, related to Figure 3

Table S4. The relative levels of transcription factors specifically activated in the eight samples, related to Figure 3

Table S5. Specific ligand-receptor activity and expression of target genes in cluster 2 in the necrotic niche, related to Figure 4

Table S6. Specific ligand-receptor activity and expression of target genes in cluster 2 in the infiltrating niche, related to Figure 4
