# Supplementary material for: Integration of multi-omics data revealed the orphan CpG islands and enhancer-dominated cis-regulatory network in glioma
Source: iScience. 2024 Sep 13;27(10):110946. doi: 10.1016/j.isci.2024.110946 (PMC11465130; doi:10.1016/j.isci.2024.110946)
Supplement: Document S1. Figures S1–S12 and Tables S2 and S7 [file mmc1.pdf]

iScience, Volume *27*

Supplemental information

Integration of multi-omics data revealed the orphan CpG islands and enhancer-dominated *cis*-regulatory network in glioma

Jiawei Yao, Penglei Yao, Yang Li, Ke He, Xinqi Ma, Qingsong Yang, Junming Jia, Zeren Chen, Shan Yu, Shuqing Gu, Kunliang Chen, Yan Zhao, Weihua Li, Guangzhi Wang, and Mian Guo

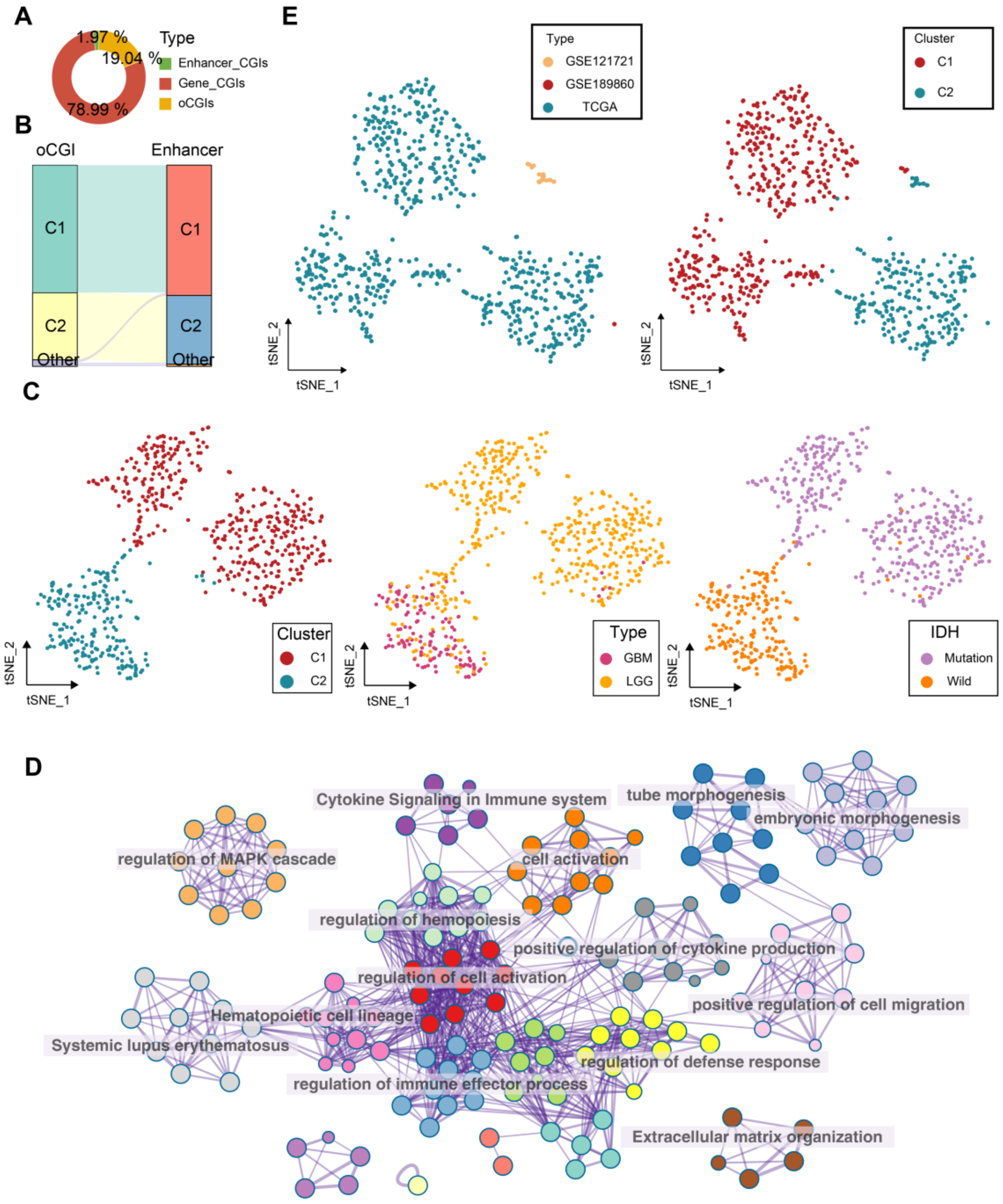

Figure S1. Identification of glioma subtypes and functional analysis. Related to Figure 1.

A. Proportion of oCGIs, enhancers, and genes in the human genome.

B. Correspondence between clustering results based on oCGIs and enhancers DNA methylation data in the TCGA cohort.

C. UMAP plot of the TCGA cohort based on oCGI DNA methylation data clustering results, and the corresponding histological type and IDH mutation status of the samples.

D. Enrichment analysis of upregulated genes in Cluster 2 based on TCGA cohort.

E. Distribution of glioma subtypes in the training set (TCGA) and test sets (GSE121721; GSE189860).

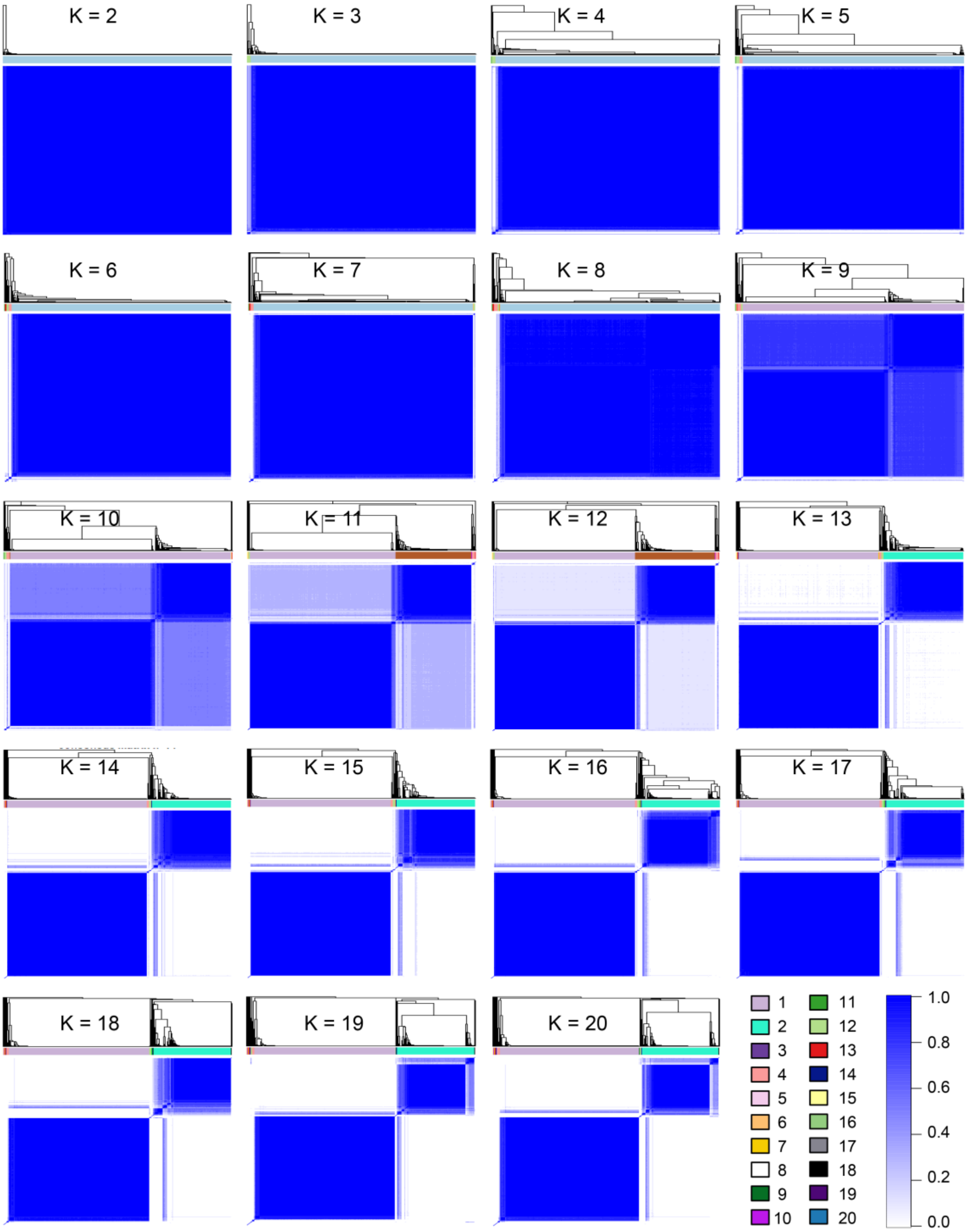

Figure S2. Consistency clustering heatmap based on TCGA oCGI methylation data. (K indicates the number of clusters) Related to Figure 1.

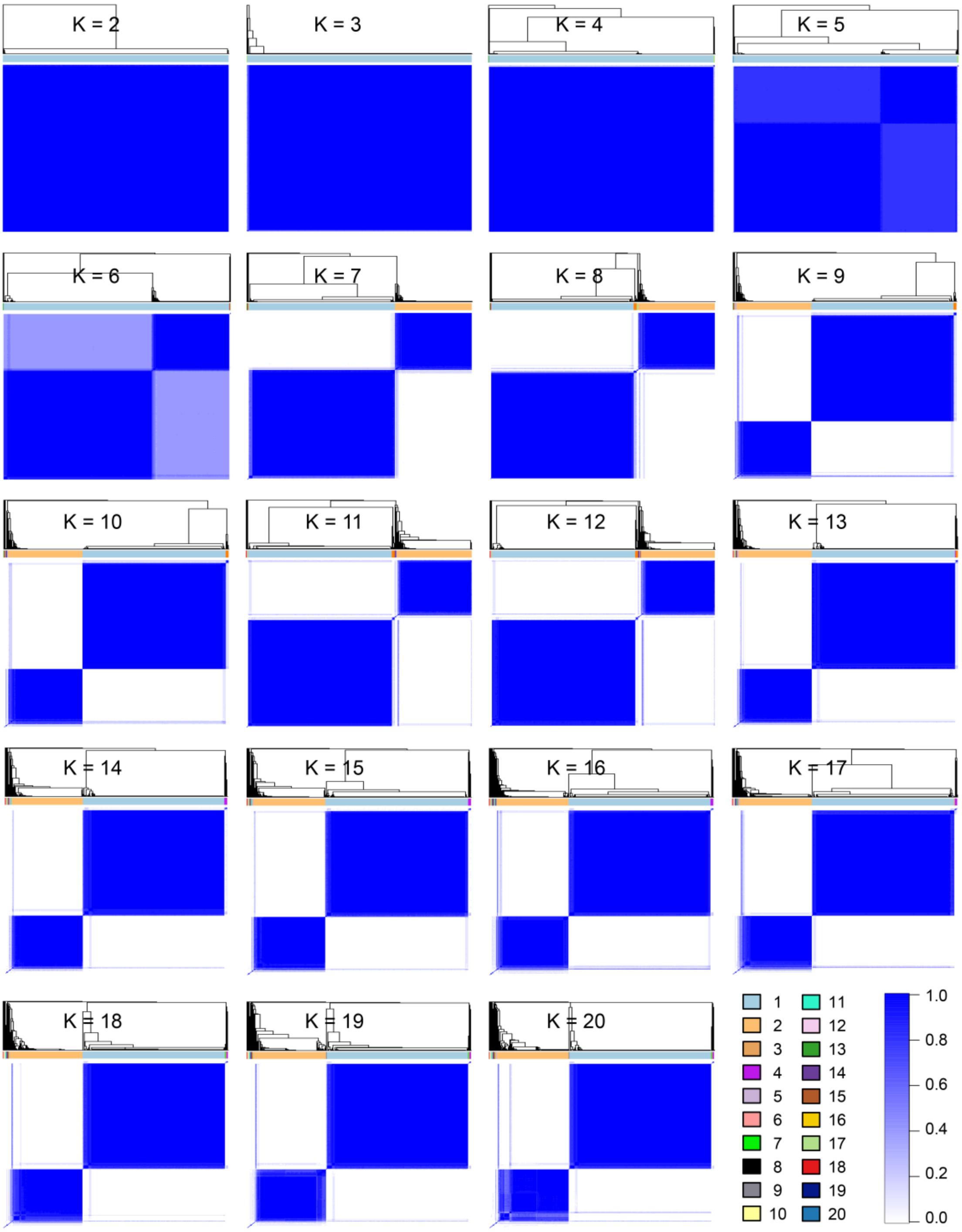

Figure S3. Consistency clustering heatmap based on TCGA enhancer methylation. (K indicates the number of clusters) Related to Figure 1.

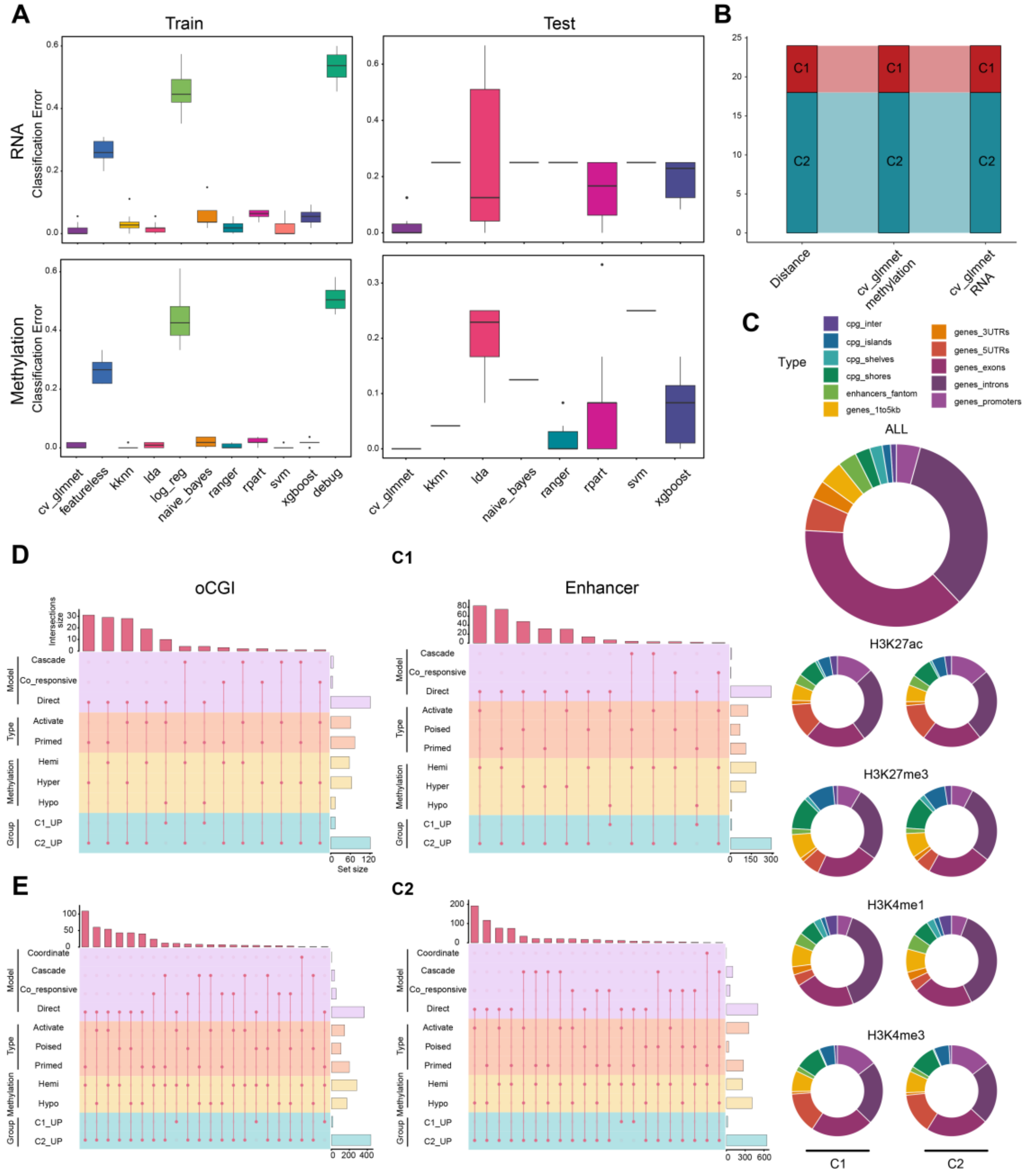

Figure S4. Functional status of oCGIs and enhancers in glioma subtypes. Related to Figure 2.

A. Classification error of 11 machine learning algorithms applied to build classifiers for oCGI RNA expression and methylation levels.

B. Distances between samples and the correlations between 3 classifiers after applying cv_glmnet to oCGIs DNA methylation levels and RNA expression.

C. Distribution of histone modifications H3K27ac, H3K27me3, H3K4me1, and H3K4me3 in the genomes of Cluster 1 and Cluster 2.

D. and E. Functional states of oCGIs and enhancers in the regulatory models of the triplets in which oCGIs or enhancers play a dominant role, and the relationship between DNA methylation levels and gene expression in Cluster 1 and Cluster 2.

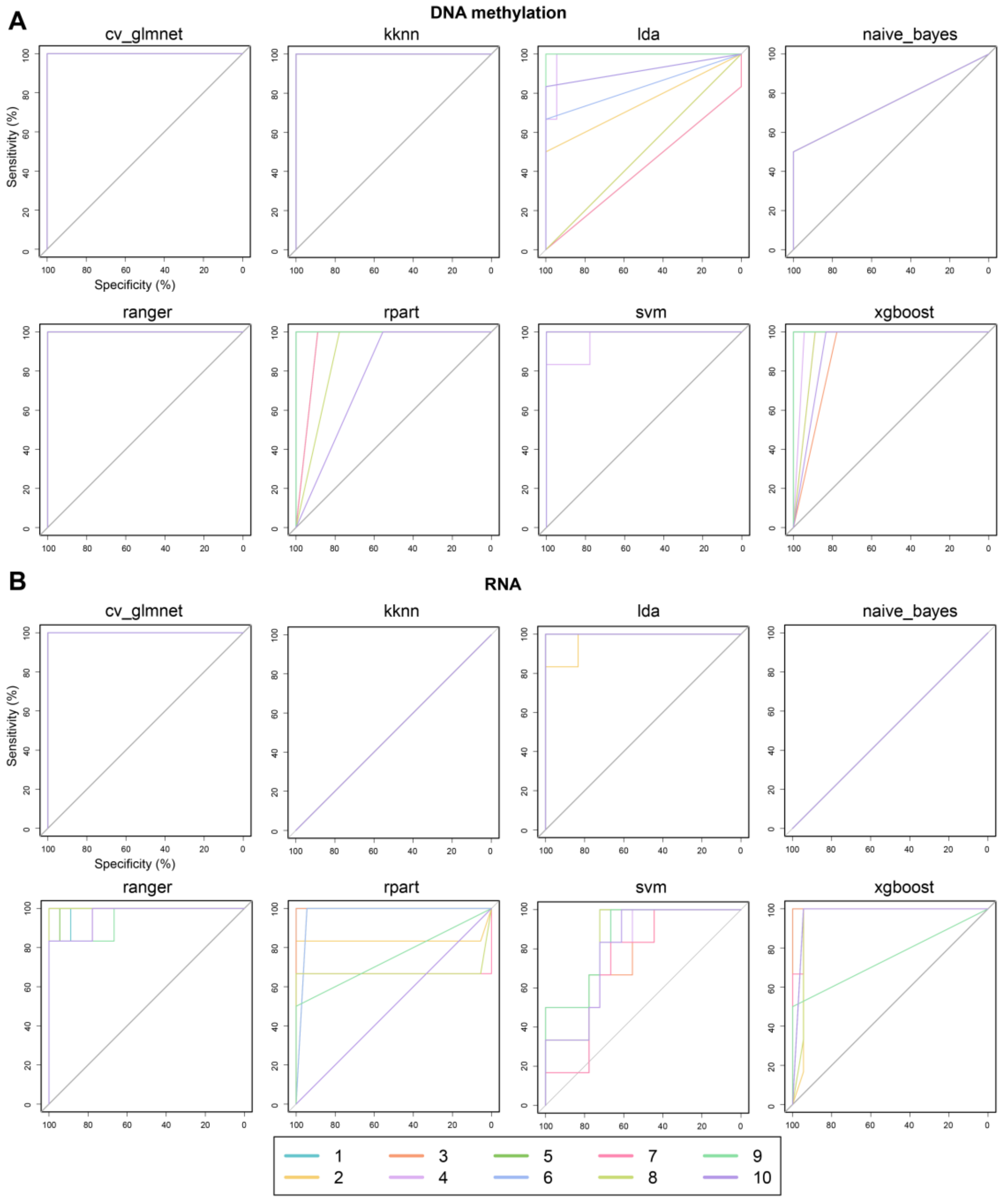

Figure S5. ROC curves for machine learning. Related to Figure 2.

A. and B. ROC curves for 10 models of 8 algorithms based on oCGIs DNA methylation and RNA expression. (Each color in the legend corresponds to a different model of the same algorithm.)

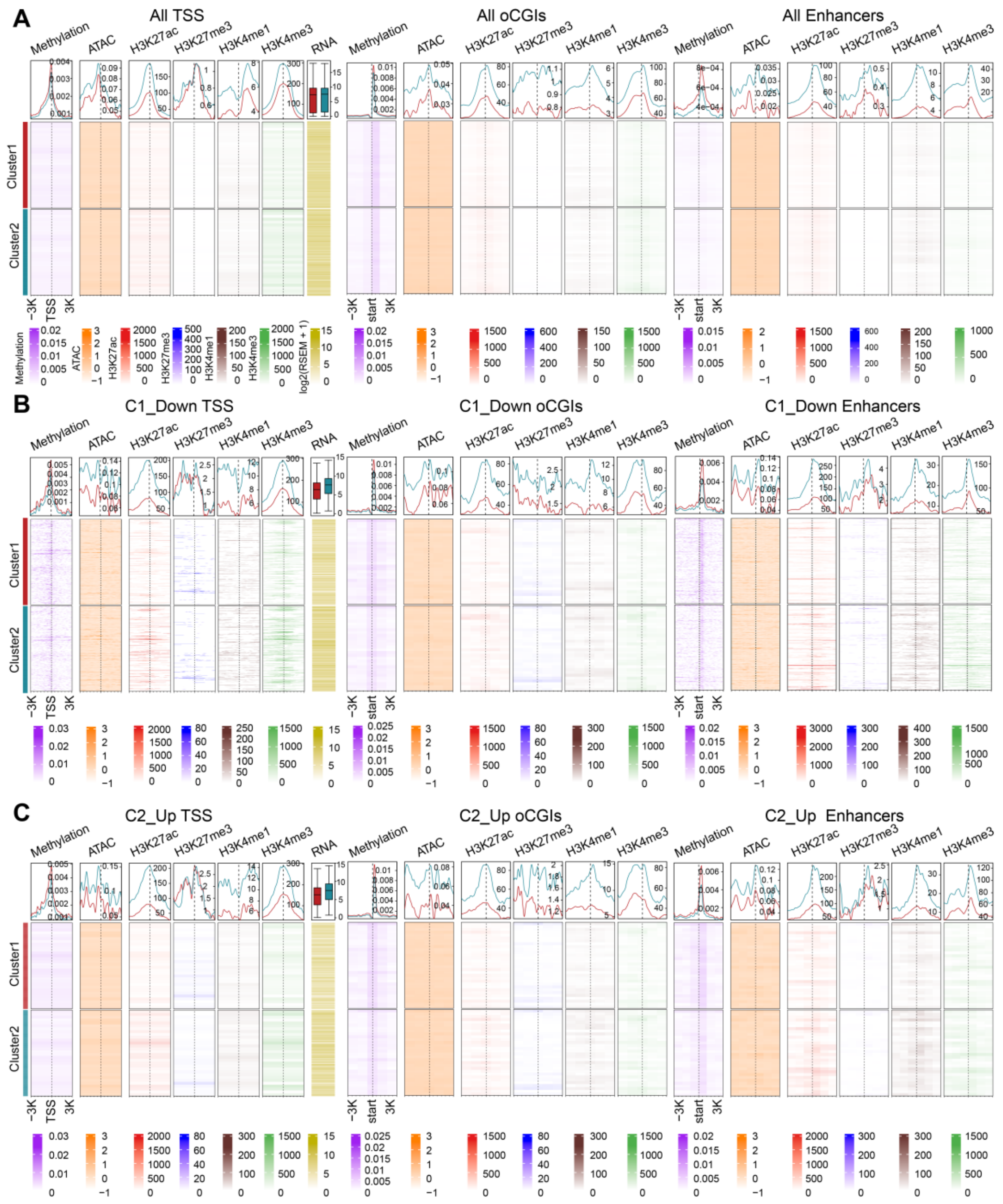

Figure S6. Chromatin modifications in the cis-regulatory models in which oCGIs or enhancers play a dominant role. Related to Figure 2.

A. Methylation, ATAC, and 4 histone modifications (H3K27ac, H3K27me3, H3K4me1, and H3K4me3) profiles for all TSS, oCGIs, and enhancers in Cluster 1 and Cluster 2.

B. Methylation, ATAC, and 4 histone modification profiles for downregulated components of cis-regulatory models of Cluster 1 in Cluster 1 and Cluster 2.

C. Methylation, ATAC, and 4 histone modifications profiles for the upregulated components of cis-regulatory models of Cluster 2 in Cluster 1 and Cluster 2.

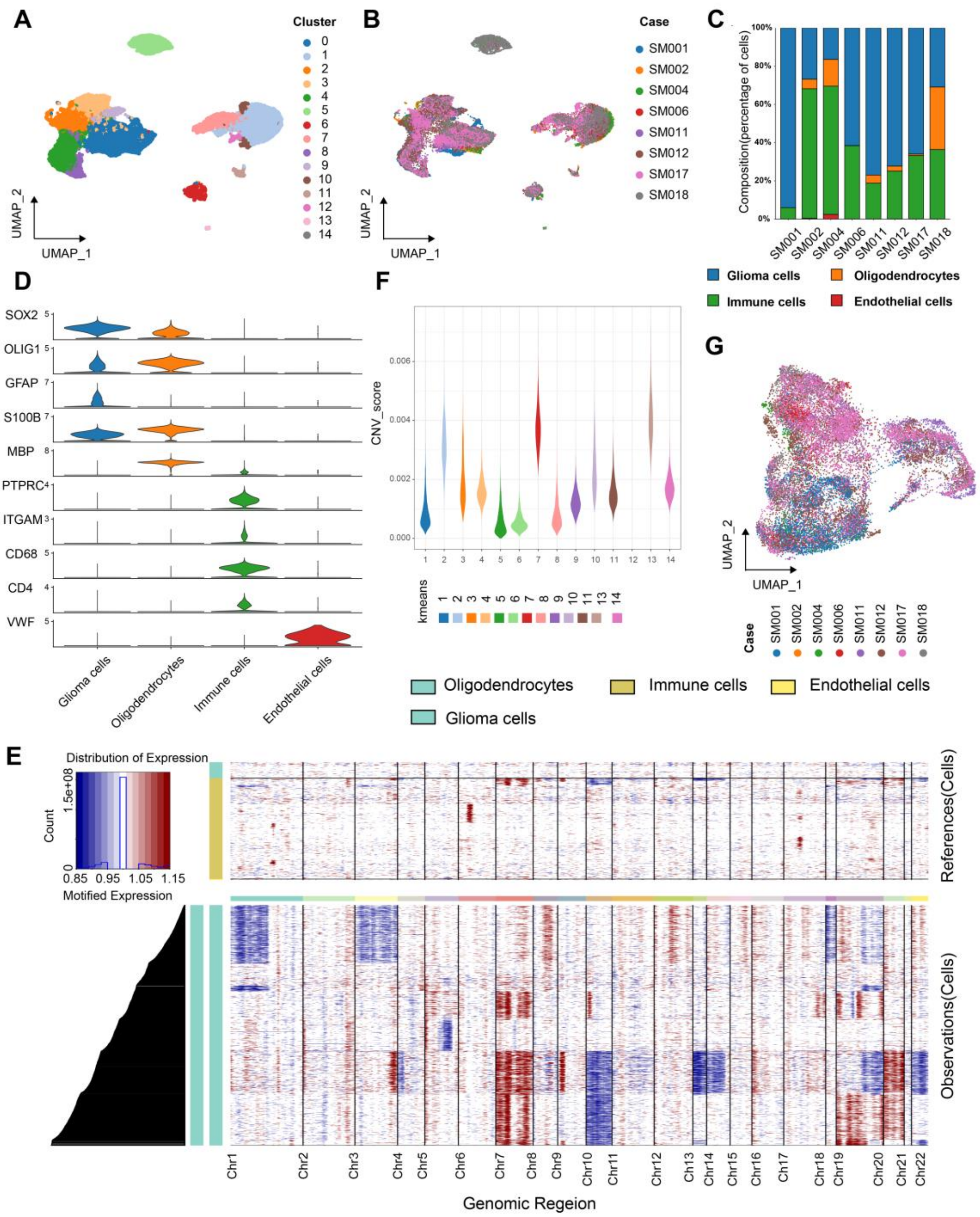

Figure S7. Overview of scRNA-seq. Related to Figure 3.

A. UMAP of Seurat clusters from 8 gliomas.

B. UMAP of sample origin from 8 gliomas.

C. Cellular composition of each glioma sample.

D. The violin plots of marker genes for different cell types.

E. The inference of copy number variation (CNV) from scRNA-seq

F. The violin plots of k-means CNV clustering results.

G. UMAP of sample origin for tumor cells.

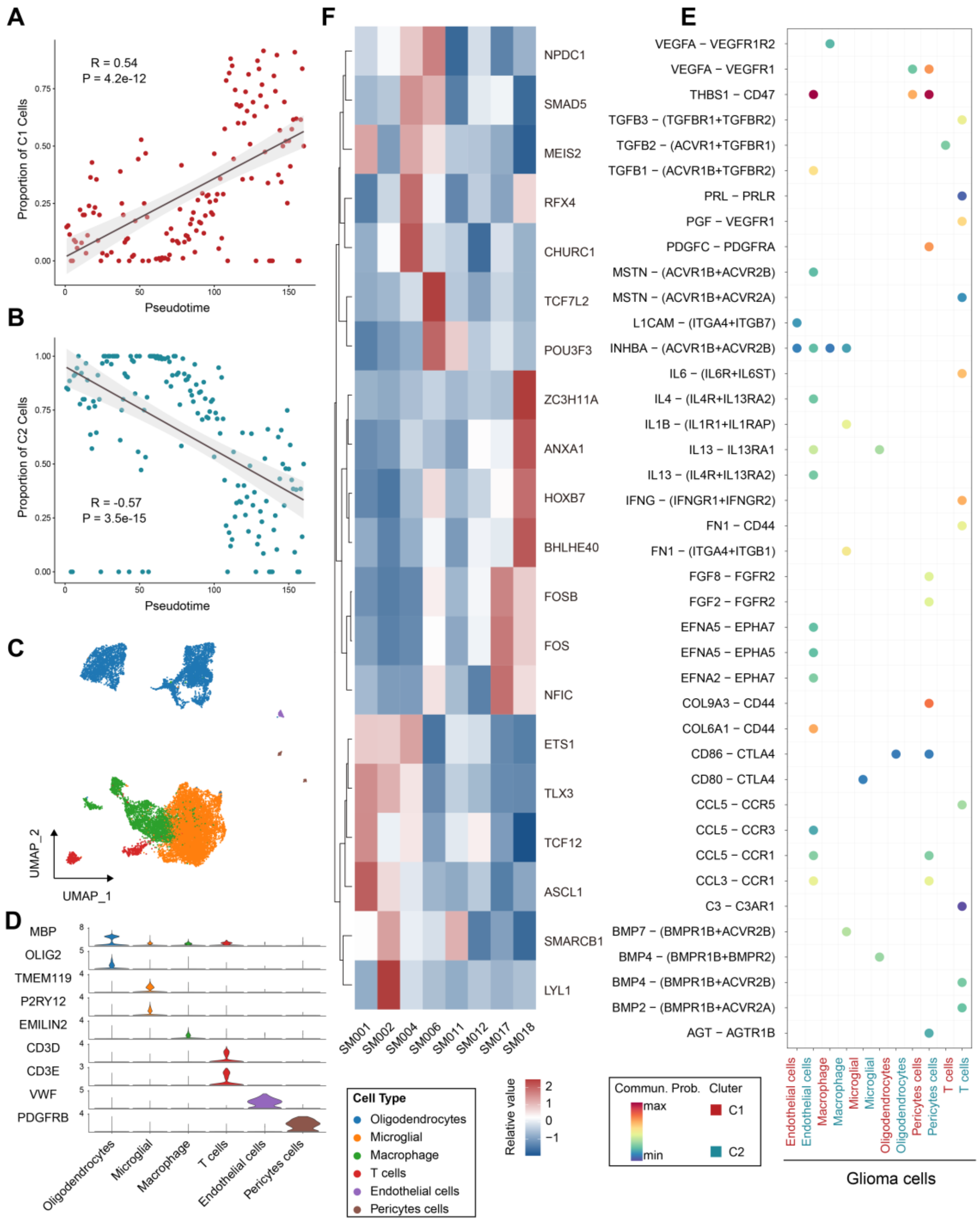

Figure S8. Transcriptional regulation and cell communication in glioma cells. Related to Figure 3.

A. and B. Changes in the proportion of tumor cells from Cluster 1 and Cluster 2 along the differentiation trajectory.

C. Cell annotation of non-tumor cells in scRNA-seq.

D. The violin plots of marker genes of non-tumor cells.

E. Intensity of different ligand-receptor interactions between glioma cells and other components of the tumor microenvironment in the two glioma subtypes.

F. The relative expression levels of transcription factors specifically activated in the eight samples.

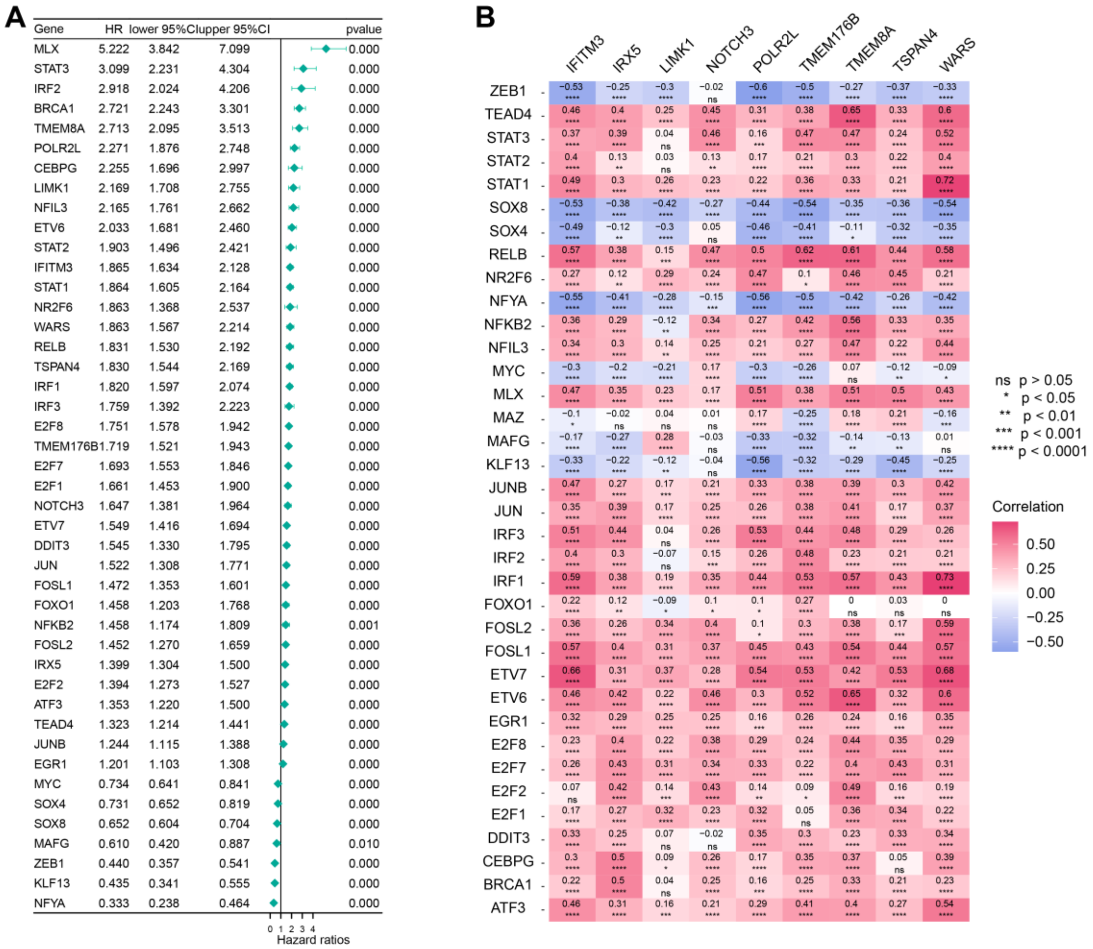

Figure S9

A. Univariate cox regression of TFs based on TCGA. Related to Figure 3.

B. Co-expression heatmap between TFs and target genes based on TCGA (Rows represent transcription factors, and columns represent target genes.)

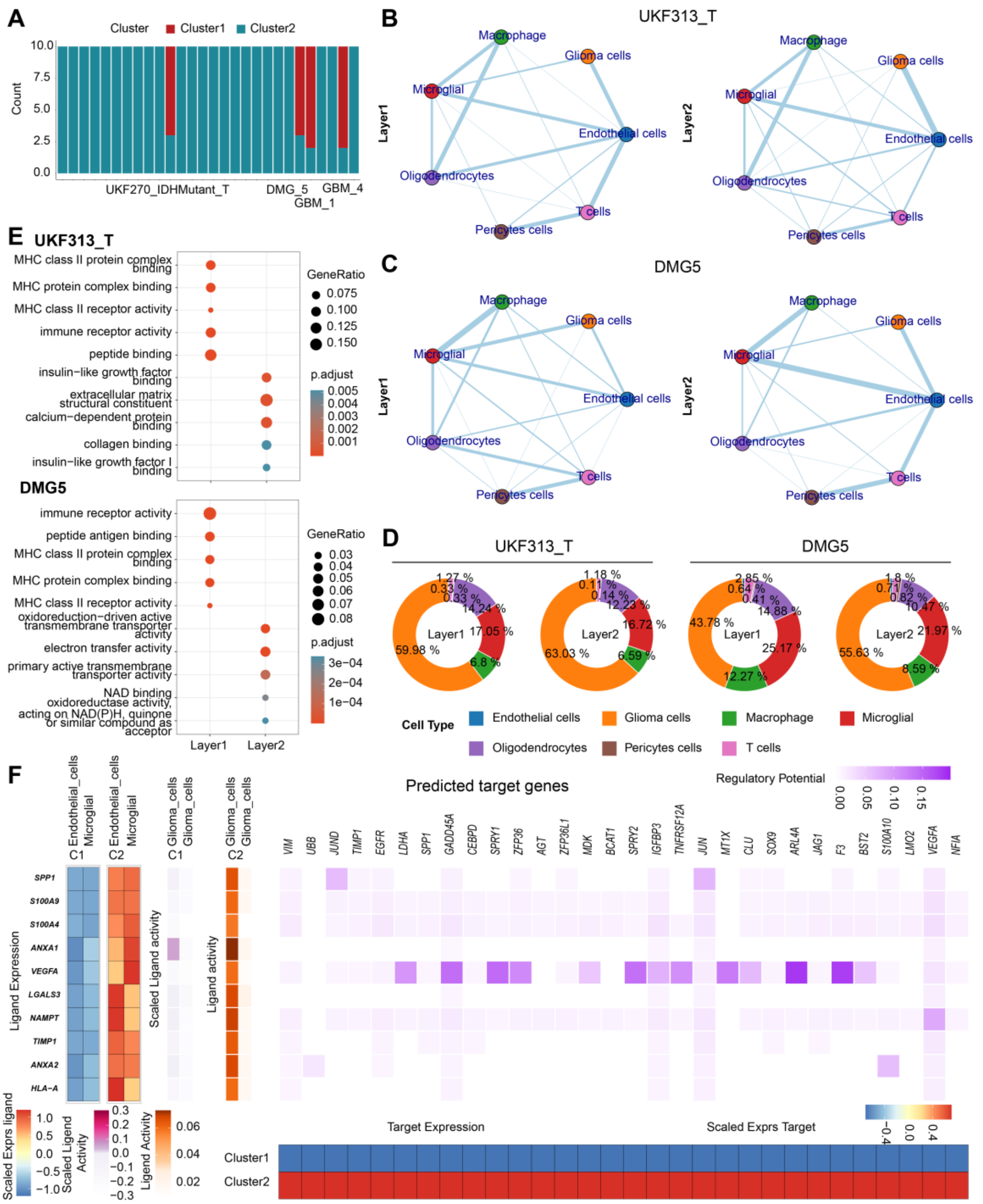

Figure S10. Spatial transcriptomic features of the necrotic niche. Related to Figure 4.

A. Glioma subtype identification based on oCGI RNA classifier for stRNA-seq pseudobulk of 29 glioma samples.

B. and C. Cell co-localization analysis in the necrotic niche for UKF313_T (Cluster 2) and DMG5 (Cluster 1).

D. Cellular composition at different distances from the necrotic center for UKF313_T (Cluster 2) and DMG5 (Cluster 1).

E. GO analysis at different distances from the necrotic center for UKF313_T (Cluster 2) and DMG5 (Cluster 1).

F. The ligand-receptor activity and expression of target genes in Cluster 2 in the necrotic niche.

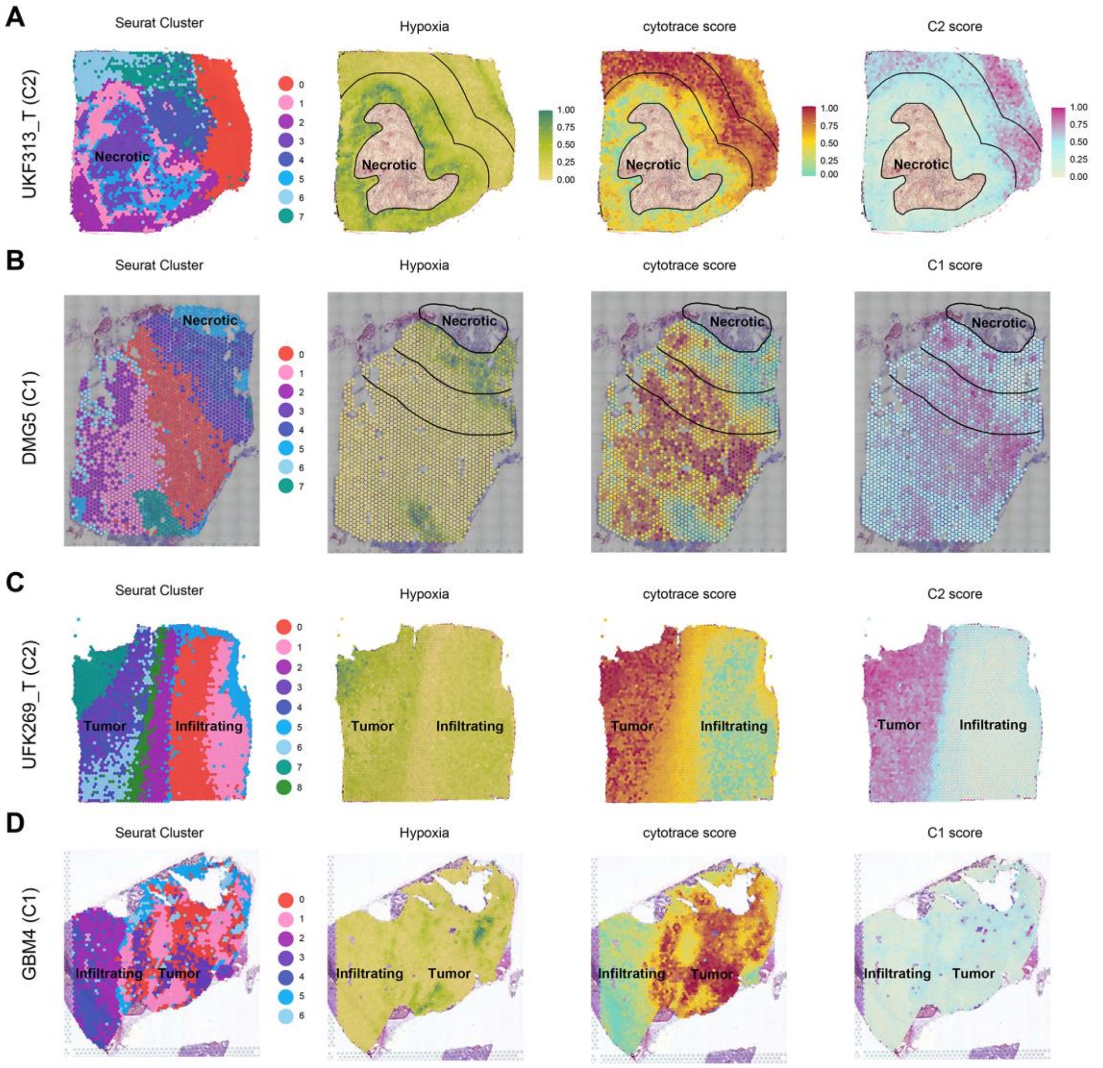

Figure S11. Distinct expression features of glioma based on stRNA-seq. Related to Figure 4.

A. and B. Distribution of Seurat clusters, hypoxia, cytotrace score, and cis score in the necrotic niche for UKF313_T (Cluster 2) and DMG5 (Cluster 1).

C. and D. Distribution of Seurat clusters, hypoxia, cytotrace score, and cis score in infiltrating niche for UFK269_T (Cluster 2) and GBM4 (Cluster 1).

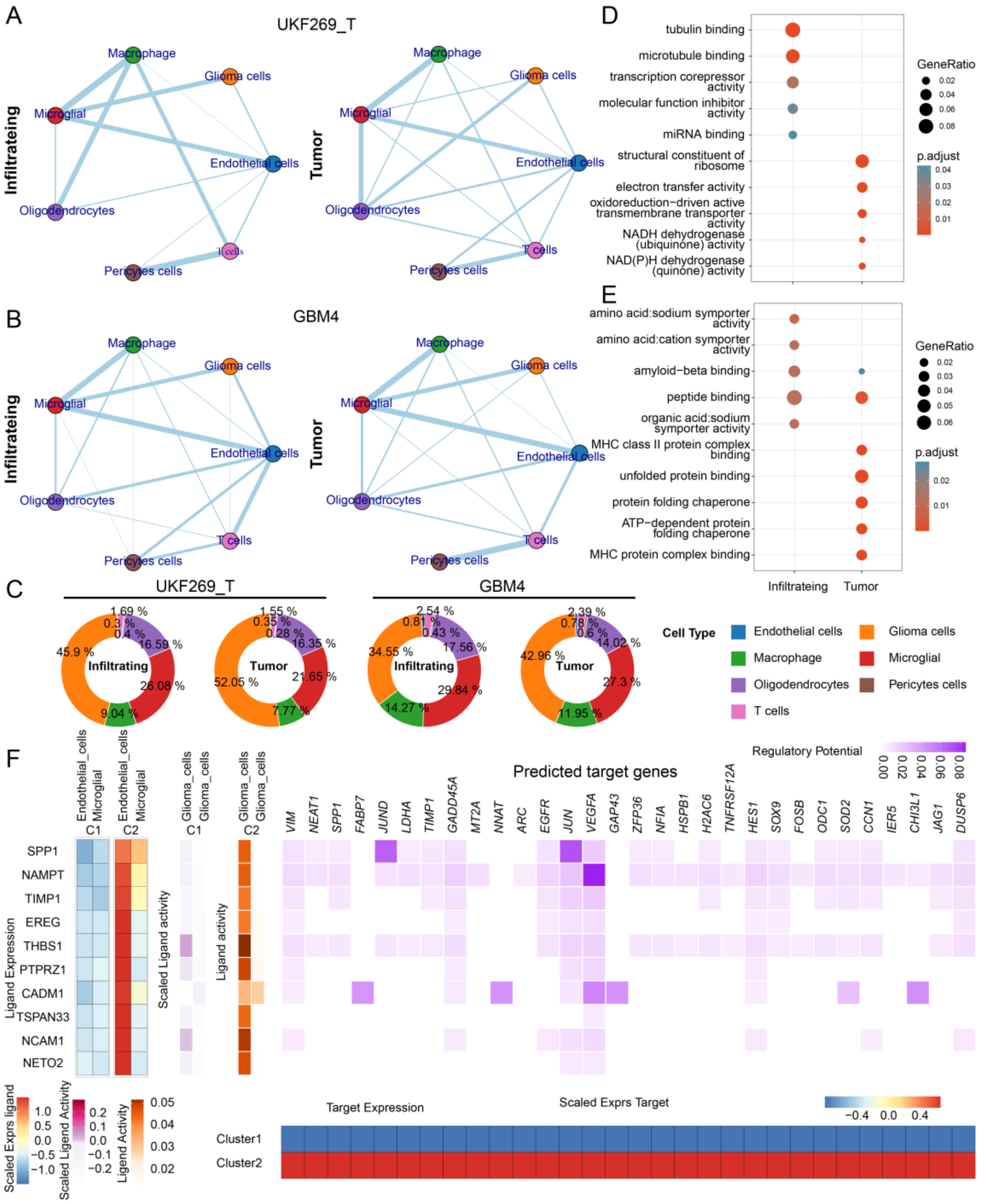

Figure S12. Spatial transcriptomic features of the infiltrating niche. Related to Figure 4.

A. and B. Cell co-localization analysis in the infiltrating niche for UFK269_T (Cluster 2) and GBM4 (Cluster 1).

C. Cellular composition in the tumor and infiltrating regions for UFK269_T (Cluster 2) and GBM4 (Cluster 1).

D. and E. GO analysis in the tumor and infiltrating regions for UFK269_T (Cluster 2) and GBM4 (Cluster 1).

F. The ligand-receptor activity and expression of target genes in Cluster 2 in the infiltrating niche.

Table S2 Target genes of cis-regulatory models in two glioma subtypes. Related to Figure 3.

Cluster1_Gene	Cluster2_Gene
ISG15	SDF4
VWA1	PUSL1
NADK	CITED4
NECAP2	PPCS
TMEM50A	PPIH
SEPN1	FGFRL1
MRPS15	MRPL36
DNALI1	FOXD1
RRM2	MICALL2
TRIB2	LFNG
INHBB	LIMK1
FGFRL1	PARP12
PSMG3	TMEM176B
TTYH3	TMEM176A
GRB10	IFITM3
BCL7B	CD151
LIMK1	POLR2L
RFC2	TSPAN4
PCOLCE	MDK
TMEM176B	WARS
PLAU	CDCA4
IFITM3	RHBDF1
IRF7	TMEM8A
POLR2L	TNFRSF12A
TSPAN4	GINS2
UNC93B1	NXT1
RAD51AP1	CHAF1A
CD9	NOTCH3
UNG	WTIP
WARS	IRX5
CDCA4	LRRC61
RHBDF1	EN2
HN1L	N6AMT2
MMP2	
GINS2	
NXT1	
SBNO2	
WTIP	
IRX5	
LRRC61	

EN2	

Table S7. Sequences of primers for PCR. Related to Figure 6.	
oCGI primer-F	GCCCCGTGAGGCAGGCGTGGA
oCGI primer-R	CCAAACTCGGGCCTCGAGCAG
POLR2L-F	ACACCGAGGGGGATGCGCTGG
POLR2L-R	GGCGGCAGCAGTAGCGCTTCA
SOX2-F	GCTACAGCATGATGCAGGACCA
SOX2-R	TCTGCGAGCTGGTCATGGAGTT
CD133-F	GCAATCTCCCTGTTGGTGA
CD133-R	CCAGTTTCCGACTCCTTTTG
